# Asciminib monotherapy in patients with CML-CP without *BCR::ABL1* T315I mutations treated with at least two prior TKIs: 4-year phase 1 safety and efficacy results

**DOI:** 10.1038/s41375-023-01860-w

**Published:** 2023-03-22

**Authors:** Michael J. Mauro, Timothy P. Hughes, Dong-Wook Kim, Delphine Rea, Jorge E. Cortes, Andreas Hochhaus, Koji Sasaki, Massimo Breccia, Moshe Talpaz, Oliver Ottmann, Hironobu Minami, Yeow Tee Goh, Daniel J. DeAngelo, Michael C. Heinrich, Valle Gómez-García de Soria, Philipp le Coutre, Francois-Xavier Mahon, Jeroen J. W. M. Janssen, Michael Deininger, Naranie Shanmuganathan, Mark B. Geyer, Silvia Cacciatore, Fotis Polydoros, Nithya Agrawal, Matthias Hoch, Fabian Lang

**Affiliations:** 1 Memorial Sloan Kettering Cancer Center, New York, NY USA; 2 South Australian Health and Medical Research Institute and University of Adelaide, Adelaide, SA Australia; 3 Uijeongbu Eulji Medical Center, Geumo-dong, Uijeongbu-si, South Korea; 4 Adult Hematology and INSERM CIC1427, Hôpital Saint-Louis, Paris, France; 5 Georgia Cancer Center, Augusta, GA USA; 6 Universitätsklinikum Jena, Jena, Germany; 7 The University of Texas MD Anderson Cancer Center, Houston, TX USA; 8 Department of Translational and Precision Medicine-Az., Policlinico Umberto I-Sapienza University, Rome, Italy; 9 Division of Hematology-Oncology, University of Michigan Rogel Cancer Center, Ann Arbor, MI USA; 10 Cardiff University, Cardiff, UK; 11 Kobe University Hospital, Kobe, Japan; 12 Department of Haematology, Singapore General Hospital, Bukit Merah, Singapore; 13 Dana-Farber Cancer Institute, Boston, MA USA; 14 Department of Medicine, Division of Hematology and Oncology, Portland VA Health Care System and Oregon Health & Science University, Knight Cancer Institute, Portland, OR USA; 15 Hospital de la Princesa, Madrid, Spain; 16 Charité–Universitätsmedizin Berlin, Berlin, Germany; 17 Department of Hematology, Institut Bergonié, Bordeaux, France; 18 VU University Medical Center, Amsterdam, The Netherlands; 19 Versiti Blood Research Institute, Milwaukee, WI USA; 20 Royal Adelaide Hospital, Adelaide, SA Australia; 21 Novartis Pharma AG, Basel, Switzerland; 22 Department for Hematology/Oncology, Goethe University Hospital, Frankfurt am Main, Germany

**Keywords:** Drug development, Chronic myeloid leukaemia

## Abstract

Asciminib is approved for patients with Philadelphia chromosome–positive chronic-phase chronic myeloid leukemia (CML-CP) who received ≥2 prior tyrosine kinase inhibitors or have the T315I mutation. We report updated results of a phase 1, open-label, nonrandomized trial (NCT02081378) assessing the safety, tolerability, and antileukemic activity of asciminib monotherapy 10–200 mg once or twice daily in 115 patients with CML-CP without T315I (data cutoff: January 6, 2021). After ≈4-year median exposure, 69.6% of patients remained on asciminib. The most common grade ≥3 adverse events (AEs) included increased pancreatic enzymes (22.6%), thrombocytopenia (13.9%), hypertension (13.0%), and neutropenia (12.2%); all-grade AEs (mostly grade 1/2) included musculoskeletal pain (59.1%), upper respiratory tract infection (41.7%), and fatigue (40.9%). Clinical pancreatitis and arterial occlusive events (AOEs) occurred in 7.0% and 8.7%, respectively. Most AEs occurred during year 1; the subsequent likelihood of new events, including AOEs, was low. By data cutoff, among patients without the indicated response at baseline, 61.3% achieved *BCR::ABL1* ≤ 1%, 61.6% achieved ≤0.1% (major molecular response [MMR]), and 33.7% achieved ≤0.01% on the International Scale. MMR was maintained in 48/53 patients who achieved it and 19/20 who were in MMR at screening, supporting the long-term safety and efficacy of asciminib in this population.

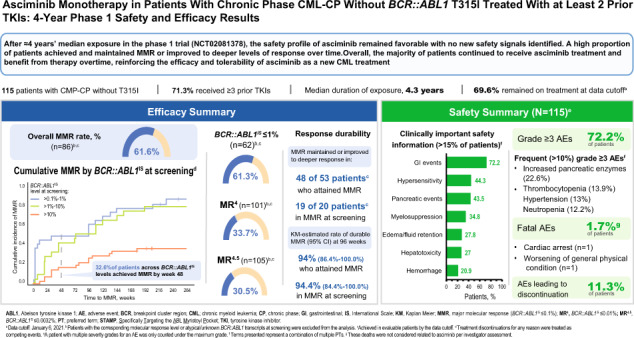

## Introduction

Tyrosine kinase inhibitors (TKIs) targeting the ABL and BCR::ABL1 adenosine triphosphate (ATP) binding sites have significantly extended the lives of patients with chronic myeloid leukemia (CML) [1–5]. However, intolerance and resistance to ATP-competitive TKIs, resulting in decreased quality of life and increased risk of progressive disease (PD), remain challenges [3–6]. ATP-competitive TKIs may have off-target effects from lack of specificity for BCR::ABL1 that can be associated with long-term safety risks and treatment discontinuation [1, 7–13]. Treatment resistance may result from emergent BCR::ABL1 mutations that are sensitive to only specific TKIs, including the T315I mutation, which confers resistance to almost all approved TKIs [2–4, 14].

As patients without satisfactory treatment outcomes advance through successive lines of TKIs, treatment failure rates increase, survival rates decrease [13, 15, 16], and many do not achieve optimal responses [1, 2, 10, 17–24]. New treatment options with improved antileukemic activity and long-term tolerability are needed for patients with resistance or intolerance to multiple TKIs.

Asciminib is the first BCR::ABL1 inhibitor that specifically targets the ABL myristoyl pocket (STAMP), allosterically restoring inhibition of the ABL1 kinase [25–28]. Unlike ATP-competitive TKIs, asciminib maintains activity against most ATP-binding site mutations, including T315I [25–27]. Asciminib’s target selectivity and specificity were predicted to minimize off-target effects, potentially reducing AEs in patients requiring long-term therapy [25–27, 29]. Its high potency may also drive rapid, sustained, and deeper molecular responses [25–27]; in the ASCEMBL trial, deeper, faster responses were achieved with asciminib versus bosutinib [28, 30]. Asciminib was approved in the US in 2021, with subsequent approvals worldwide, and was added to National Comprehensive Cancer Network guidelines as a new option for adults with Philadelphia chromosome–positive (Ph+) chronic-phase CML (CML-CP) previously treated with ≥2 TKIs or who have the T315I mutation [2, 31]. This approval was supported by results from the randomized phase 3 ASCEMBL trial (NCT03106779) and cohorts of patients in the current phase 1 trial (NCT02081378) receiving asciminib monotherapy, including heavily pretreated patients with Ph+ CML-CP/accelerated phase (AP) with or without T315I [28, 29, 31].

Prior analysis from this phase 1 trial after a median follow-up of 14 months first provided safety and efficacy data for asciminib in patients with CML-CP/AP with or without T315I [29]. Among patients with CML-CP without T315I, major molecular response (MMR; *BCR::ABL1* ≤ 0.1% on the International Scale [IS]) was achieved or maintained by 12 months in 44 of 91 evaluable patients (48%) (median response duration, >61 weeks), whereas 4 patients lost MMR (1 with MMR at baseline). Of 51 patients, 14 (27%) with *BCR::ABL1*^IS^ > 1% at baseline achieved MMR by 12 months. Updated results from a cohort of patients with T315I-mutated CML-CP will be reported separately. Here we report updated safety and efficacy results from this trial in patients with CML-CP without T315I who were treated with asciminib monotherapy over a median duration of exposure of ≈4 years.

## Methods

### Study oversight

The study was designed collaboratively by the sponsor (Novartis Pharmaceuticals) and study investigators. The sponsor collected and analyzed data in conjunction with the authors. All authors contributed to the development and writing of the manuscript and vouch for the accuracy and completeness of the data and the study’s fidelity to the protocol.

### Study design

The methods of this open-label, nonrandomized, first-in-human study of asciminib (Supplementary Fig. S1) have been described in detail elsewhere [29]. This analysis focused on the cohort of patients (*n* = 115) with Ph+ CML-CP without T315I at screening who received asciminib monotherapy at varying doses twice daily (10–200 mg) or once daily (80–200 mg) in the dose-escalation or -expansion parts of this study. Patients were ≥18 years of age; had hematologic, cytogenetic, or molecular evidence of disease that was relapsed or refractory to ≥2 prior TKIs; or were intolerant of ≥2 prior TKIs (per European LeukemiaNet 2009 recommendations [32]).

The primary objective was to determine the maximum tolerated dose and/or recommended dose for the expansion of asciminib monotherapy. Secondary objectives included assessing safety, tolerability, preliminary antileukemic activity, and pharmacokinetic profile in plasma of asciminib. Additional details are in the Supplementary Methods.

### Study assessments

AEs were coded using the Medical Dictionary for Regulatory Activities version 23.1 and graded according to the Common Terminology Criteria for Adverse Events version 4.03. Molecular response was assessed using real-time, quantitative, reverse-transcriptase polymerase chain reaction. Results were reported as the ratio of *BCR::ABL1* to *ABL1* on the IS [33]. *BCR::ABL1* mutational analyses were performed using Sanger sequencing. Molecular and mutational assessments were performed centrally by ICON (Portland, OR, USA).

### Statistical analyses

The data cutoff date was January 6, 2021, and all patients who received ≥1 study drug dose were included. The MMR rate by each time point was defined as the proportion of MMR-evaluable patients (i.e., those not in MMR and without atypical *BCR::ABL1* transcripts at screening) who had achieved MMR by that time point. Rates of *BCR::ABL1*^IS^ ≤ 1% or deep molecular response (DMR; MR^4^ [*BCR::ABL1*^IS^ ≤ 0.01%] and MR^4.5^ [*BCR::ABL1*^IS^ ≤ 0.0032%]) were calculated similarly. Event-free survival (EFS) was estimated using the Kaplan–Meier method, with events defined as treatment discontinuation due to AEs, on-treatment progression to AP/blast crisis (BC), and on-treatment death for any reason. An EFS analysis that included *BCR::ABL1*^IS^ > 10% at 6 months and >1% at ≥12 months as events is reported in the Supplementary Material. Progression-free survival (PFS) and overall survival (OS) were not analyzed because patients were not followed after treatment discontinuation.

## Results

### Patients

**Fig. 1:**
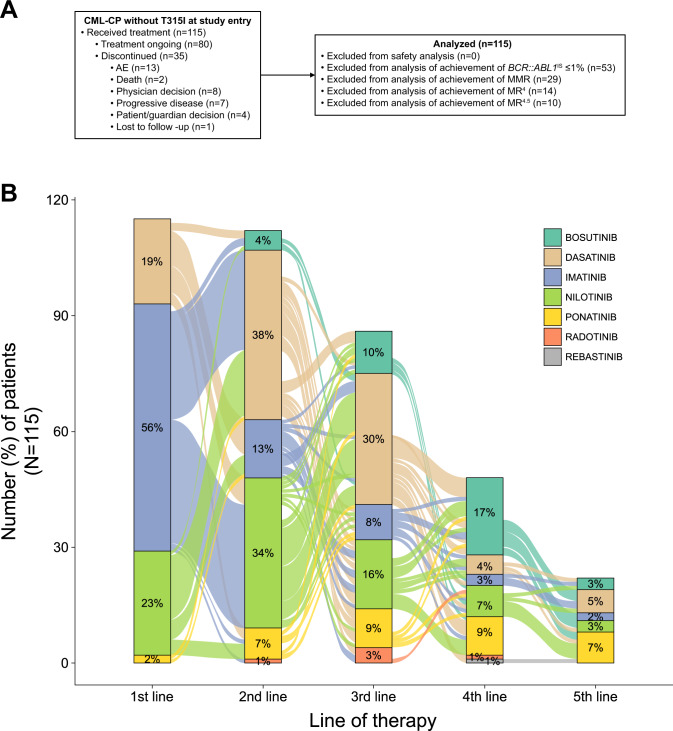
Patient disposition as of the data cutoff (January 6, 2021) and history of prior TKIs. **A** Disposition of patients with CML-CP without *BCR::ABL1* T315I mutations who received asciminib monotherapy. **B** History of prior TKIs. AE adverse event, CML chronic myeloid leukemia, CP chronic phase, IS International Scale, MMR major molecular response (*BCR::ABL1* ≤ 0.1% on the IS), MR^4^ *BCR::ABL1*^IS^ ≤ 0.01%, MR^4.5^ *BCR::ABL1*^IS^ ≤ 0.0032%, TKI tyrosine kinase inhibitor.

This analysis included 115 patients with CML-CP without T315I who were enrolled in the study (May 2014–October 2019) and received asciminib monotherapy (Fig. 1A, Supplementary Table S1 and Supplementary Fig. S2). At data cutoff, most patients (*n* = 80 [69.6%]) remained on study treatment; 35 patients (30.4%) had discontinued, most frequently due to AEs (*n* = 13) or physician decision (*n* = 8; mostly due to lack of efficacy). The overall median duration of exposure was 4.2 years (range, 0.04–6.55 years), and ranged from 3.8 to 4.8 years depending on the treatment line (Supplementary Fig. S3); 99 patients (86.1%) were exposed for ≥48 weeks and 88 (76.5%) for ≥96 weeks. Two patients (1.7%) died on treatment (defined as on treatment or within 30 days of the last study drug dose), and deaths were unrelated to asciminib per investigator assessment (Fig. 1A and Supplementary Material): 1 patient died due to cardiac arrest and had a history of suspected contributory comorbidities (e.g., systemic scleroderma and ischemic heart disease), and another patient with a history of bladder cancer and urostomy died due to worsening of general physical condition.

Patients were heavily pretreated; the majority (71.3%) received ≥3 prior TKIs (Table 1). Three patients (2.6%) who were positive for T315I on enrollment had received one prior TKI, per eligibility criteria; mutation was not confirmed by the central laboratory. Imatinib was the first-line TKI for 56% of patients (Fig. 1B); dasatinib (38%) and nilotinib (34%) were the most frequent second-line therapies. TKI use patterns were more complex in the third line and beyond. Additional baseline demographics are in Supplementary Table S2. At screening, *BCR::ABL1* mutations were detected in 12 patients (10.4%), with 2 (1.7%) having multiple mutations; 29 patients (25.2%) were not evaluable (*n* = 10) or had low levels of *BCR::ABL1* leading to lack of amplification (*n* = 19) (Table 1 and Supplementary Table S3).

**Table 1:** Baseline patient demographics and clinical characteristics.

Variable	All patients (*N* = 115)
Age, median (range), years	56.0 (25–88)
Age ≥65 years, *n* (%)	30 (26.1)
Sex, *n* (%)	
Male	60 (52.2)
Female	55 (47.8)
ECOG performance status, *n* (%)	
0	87 (75.7)
1	26 (22.6)
2	2 (1.7)
No. of prior TKIs, *n* (%)	
1	3 (2.6)^a^
2	30 (26.1)
3	41 (35.7)
4	32 (27.8)
≥5	9 (7.8)
Prior TKIs, *n* (%)	
Dasatinib	98 (85.2)
Nilotinib	89 (77.4)
Imatinib	85 (73.9)
Bosutinib	45 (39.1)
Ponatinib	36 (31.3)
Radotinib	6 (5.2)
Rebastinib	1 (0.9)
Time since diagnosis, median (range), years	4.41 (0.77–26.55)
*BCR::ABL1*^IS^ at screening, *n* (%)	
>10%	41 (35.7)
>1% to ≤10%	21 (18.3)
≤1%	44 (38.3)
>0.1% to ≤1%	24 (20.9)
>0.01% to ≤0.1%	15 (13.0)
≤0.01%	5 (4.3)
>0.0032% to ≤0.01%	4 (3.5)
≤0.0032%	1 (0.9)
Atypical/unknown transcripts, *n* (%)	9 (7.8)
p190 (e1a2)	5 (4.3)
e1a3	1 (0.9)
e19a2	1 (0.9)
Novel variant	1 (0.9)
Unknown, not detected	1 (0.9)
*BCR::ABL1* mutations at screening, *n* (%)	
No mutation detected	74 (64.3)
One mutation	10 (8.7)^b^
Multiple mutations	2 (1.7)
No amplification^c^	19 (16.5)
Not evaluable	10 (8.7)

*BCR::ABL1*^IS^ *BCR::ABL1* transcript levels on the International Scale, *ECOG* Eastern Cooperative Oncology Group, MR^4^ *BCR::ABL1*^IS^ ≤ 0.01%, TKI tyrosine kinase inhibitor.

^a^These three patients were enrolled as being positive for T315I based on local assessment (only had to have received one prior TKI per eligibility criteria); however, this mutation was not confirmed by the central laboratory.

^b^One patient who had an L248V mutation at screening also had an L248-K274del splice artifact at screening that was caused by the L248V mutation.

^c^*BCR::ABL1* could not be amplified due to low transcript levels.

### Safety

Treatment-emergent all-grade AEs regardless of relationship to the study drug were reported in all patients (Table 2); those reported in ≥20% were musculoskeletal pain, upper respiratory tract infection, fatigue, increased pancreatic enzymes (mostly asymptomatic, as reported in detail below), abdominal pain, arthralgia, headache, diarrhea, nausea, hypertension, rash, vomiting, thrombocytopenia, pruritus, increased hepatic enzymes, and dizziness. Grade ≥3 AEs reported in ≥10% of patients were increased pancreatic enzymes, thrombocytopenia, hypertension, and neutropenia.

**Table 2:** Treatment-emergent AEs regardless of relationship to study drug (occurring in ≥10% of patients) up to data cutoff.

Event, *n* (%)^a,b^	All patients (*N* = 115)	
	All grades	Grade ≥3
≥1 AE	115 (100)	83 (72.2)
Musculoskeletal pain	68 (59.1)	6 (5.2)
Upper respiratory tract infection	48 (41.7)	0
Fatigue	47 (40.9)	2 (1.7)
Increased pancreatic enzymes	46 (40.0)	26 (22.6)
Abdominal pain	43 (37.4)	1 (0.9)
Arthralgia	42 (36.5)	3 (2.6)
Headache	38 (33.0)	3 (2.6)
Diarrhea	35 (30.4)	0
Nausea	33 (28.7)	2 (1.7)
Hypertension	32 (27.8)	15 (13.0)
Rash	32 (27.8)	0
Vomiting	30 (26.1)	3 (2.6)
Thrombocytopenia	29 (25.2)	16 (13.9)
Pruritus	26 (22.6)	1 (0.9)
Increased hepatic enzymes	24 (20.9)	4 (3.5)
Dizziness	23 (20.0)	0
Dyslipidemia	22 (19.1)	3 (2.6)
Constipation	22 (19.1)	0
Cough	21 (18.3)	0
Anemia	20 (17.4)	10 (8.7)
Neutropenia	19 (16.5)	14 (12.2)
Edema	17 (14.8)	0
Lower respiratory tract infection	15 (13.0)	5 (4.3)
Dyspnea	15 (13.0)	0
Pyrexia	14 (12.2)	1 (0.9)
Increased weight	14 (12.2)	2 (1.7)
Anxiety	13 (11.3)	1 (0.9)
Decreased appetite	13 (11.3)	0
Hyperglycemia	13 (11.3)	2 (1.7)
Hyperhidrosis	13 (11.3)	0
Oropharyngeal pain	13 (11.3)	0
Depression	12 (10.4)	0
Dry eye	12 (10.4)	0
Insomnia	12 (10.4)	1 (0.9)
Noncardiac chest pain	12 (10.4)	0

*AE* adverse event, *PT* preferred term.

^a^A patient with multiple grades of severity for an event was only counted under the maximum grade.

^b^Musculoskeletal pain includes PTs pain in extremity, myalgia, back pain, bone pain, neck pain, musculoskeletal pain, musculoskeletal chest pain, and musculoskeletal discomfort. Upper respiratory tract infection includes PTs upper respiratory tract infection, nasopharyngitis, pharyngitis, and rhinitis. Fatigue includes PTs fatigue and asthenia. Increased pancreatic enzymes include PTs increased lipase and increased amylase. Abdominal pain includes PTs abdominal pain and upper abdominal pain. Hypertension includes PTs increased blood pressure and hypertension. Rash includes PTs rash and maculopapular rash. Thrombocytopenia includes PTs thrombocytopenia and decreased platelet count. Increased hepatic enzymes include PTs increased alanine aminotransferase, increased gamma-glutamyl transferase, increased aspartate aminotransferase, and transaminases increased. Dyslipidemia includes PTs increased blood cholesterol, increased blood triglycerides, hypertriglyceridemia, hypercholesterolemia, and hyperlipidemia. Anemia includes PTs anemia and decreased hemoglobin. Neutropenia includes PTs neutropenia and neutrophil count decreased. Edema includes PTs edema and peripheral edema. Lower respiratory tract infection includes PTs pneumonia and bronchitis.

**Fig. 2:**
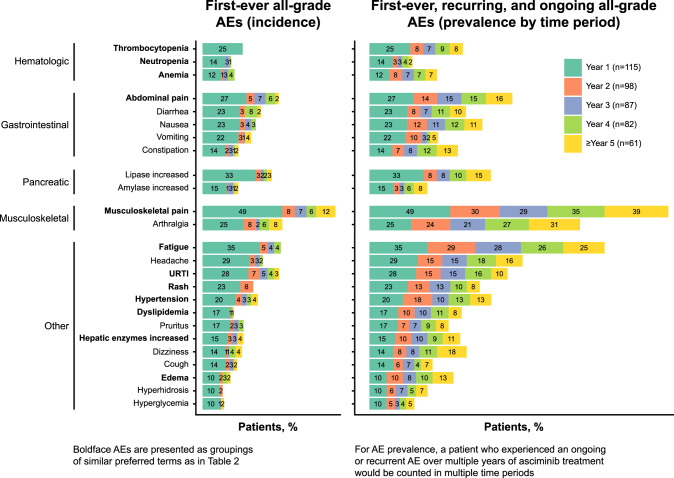
First-ever all-grade AEs (incidence) and first-ever, recurring, and ongoing all-grade AEs (prevalence by year) within individual time periods of asciminib treatment (≥10% of patients within year 1). Percentages are calculated based on the number of patients at risk of an event (left column: patients with ongoing treatment who were event free at the start of the interval; right column: patients with ongoing treatment at the start of the interval). In the left column (incidence), the number of patients at risk of an event differs from year to year, and percentages in each year should thus not be summed. A patient with multiple occurrences of an event within the same time interval was counted only once in that time interval. AE adverse event, URTI upper respiratory tract infection.

A side-by-side comparison of the first-ever occurrence of any-grade AEs (incidence) versus the persistence, recurrence, and late onset of any-grade AEs (first ever, recurring, and ongoing [prevalence]) by year shows that most AEs occurred early after treatment initiation; the likelihood of newly occurring events after the first year of treatment was low (Fig. 2). All instances of newly occurring thrombocytopenia and nearly all instances of newly occurring neutropenia, anemia, increased lipase, increased amylase, and dyslipidemia were reported in the first year. Upper respiratory tract infections and constitutional events such as musculoskeletal pain and fatigue were the only events to occur at later timepoints, were mostly grade 1/2, and did not lead to treatment discontinuation.

AEs led to treatment discontinuation in 13 patients (11.3%) (increased lipase, *n* = 4; increased amylase, pancreatitis, and thrombocytopenia, *n* = 2 each; all other AEs, *n* = 1 each) (Supplementary Table S4). In 69 patients (60.0%), AEs could be managed by dose interruption or adjustment per protocol.

Clinically important safety information (Supplementary Methods), grouped into categories, is reported in Table 3 and Supplementary Materials. The two most frequently reported categories were gastrointestinal (GI) toxicity (72.2%) and hypersensitivity (44.3%). The most frequent GI events (≥20% of patients) were abdominal pain (37.4%), diarrhea (30.4%), nausea (28.7%), and vomiting (26.1%). Grade 3 GI events were reported in 5 patients (4.3%) and did not require treatment discontinuation; dose adjustments and interruptions occurred in 7 (6.1%) and 9 (7.8%) patients, respectively. Most hypersensitivity events were low-grade skin conditions such as rash (27.8%) and urticaria (5.2%); grade 3 events occurred in 4 patients (3.5%) only. Treatment discontinuation was required in 1 patient (0.9%) (bronchospasm and rash) and dose adjustments and interruptions in 1 (0.9%) and 3 (2.6%) patients, respectively. No grade 4 events were reported.

**Table 3:** Clinically important safety information regardless of relationship to study drug (≥10% of patients).

Safety information categories (≥10% of patients), *n* (%)^a^	All patients (*N* = 115)	
	All grades	Grade ≥3
Gastrointestinal events	83 (72.2)	5 (4.3)
Hypersensitivity^b^	51 (44.3)	4 (3.5)
Pancreatic events	50 (43.5)	30 (26.1)
Pancreatitis/acute pancreatitis	8 (7.0)	4 (3.5)
Pancreatic enzyme elevations^c^	46 (40.0)	26 (22.6)
Myelosuppression^d^	40 (34.8)	23 (20.0)
Edema and fluid retention	32 (27.8)	4 (3.5)
Hepatotoxicity (clinical and laboratory events)	31 (27.0)	6 (5.2)
Clinical events^e^	4 (3.5)	1 (0.9)
Hemorrhage	24 (20.9)	4 (3.5)
Arterial occlusive events^f^	10 (8.7)	5 (4.3)

*AE* adverse event, *PT* preferred term, *TKI* tyrosine kinase inhibitor.

^a^A patient with multiple grades of severity for an AE was only counted under the maximum grade.

^b^Includes PTs allergic conjunctivitis, periorbital edema, swollen tongue, lip swelling, face edema, face swelling, drug hypersensitivity, pustular rash, allergic rhinitis, bronchospasm, rash, urticaria, maculopapular rash, dermatitis, pruritic rash, eczema, acneiform dermatitis, follicular rash, bullous dermatitis, and circulatory collapse.

^c^Includes PTs lipase increased and amylase increased.

^d^Includes PTs anemia, leukopenia, thrombocytopenia, and cytopenias affecting >1 lineage.

^e^Includes PTs ascites, liver disorder, hepatocellular injury, hepatic steatosis, and hepatic lesion.

^f^This category was included, despite not meeting the 10% threshold, because of scientific and medical interest due to class risks of other TKIs.

Pancreatic enzyme elevations were reported in 46 patients (40.0%) (grade 3, *n* = 23 [20.0%]; grade 4, *n* = 3 [2.6%]). The majority were asymptomatic and included increased lipase (*n* = 43 [37.4%]; grade 3, *n* = 21 [18.3%]; grade 4, *n* = 2 [1.7%]) and increased amylase (*n* = 22 [19.1%]; grade 3, *n* = 4 [3.5%]; grade 4, *n* = 1 [0.9%]). Pancreatic enzyme elevations led to treatment discontinuation in 4 patients (3.5%) (increased lipase, *n* = 4; increased amylase, *n* = 2) and were managed by dose adjustment in 13 (11.3%) and treatment interruption in 14 (12.2%).

Both early- and late-onset clinical pancreatitis events were reported in 8 patients (7.0%) (grade 3, *n* = 4 [3.5%]; grade 4, *n* = 0) (Supplementary Table S5 and Supplementary Fig. S4) and led to treatment discontinuation in 2 patients (1.7%) (grade 2, *n* = 1; grade 3, *n* = 1); 1 had a prior history of pancreatic steatosis and pancreatic enzyme elevations and the other had late-onset (day 505) pancreatitis. Pancreatic enzyme levels were not available for 2 patients at the time of pancreatitis events, based on investigator reporting (Supplementary Fig. S4D, E). Pancreatitis was managed by dose adjustment (*n* = 3 [2.6%]) and/or interruption (*n* = 4 [3.5%]) and resolved in all patients except one (persistent grade 2 with only radiologic findings) who had a history of acute pancreatitis and had not experienced improvement by the time of treatment discontinuation due to PD.

The most frequently reported myelosuppression events (≥10% of patients) were thrombocytopenia, anemia, and neutropenia. Thrombocytopenia was reported in 29 patients (25.2%) (grade 3, *n* = 2 [1.7%]; grade 4, *n* = 14 [12.2%]) and was associated with bleeding events in 7, including mild contusion, melena, petechiae, and grade 3 epistaxis and hematemesis (*n* = 1 each). Anemia was reported in 20 patients (17.4%), with half being grade 1 (*n* = 4 [3.5%]) or 2 (*n* = 6 [5.2%]) and the other half being grade 3. Neutropenia was reported in 19 patients (16.5%) (grades 3 and 4, *n* = 7 [6.1%] each) and was associated with infection in 2 patients (pneumonia and conjunctivitis, *n* = 1 each).

Treatment discontinuation for myelosuppression events occurred in 2 patients (1.7%); both had a previous history of and discontinued due to thrombocytopenia. These events were otherwise managed by dose adjustment (thrombocytopenia, *n* = 5 [4.3%]; anemia, *n* = 0; neutropenia, *n* = 3 [2.6%]) or treatment interruption (thrombocytopenia, *n* = 8 [7.0%]; anemia, *n* = 1 [0.9%]; neutropenia, *n* = 4 [3.5%]). Myelosuppression-related events tended to occur on treatment initiation and rarely persisted or occurred at later timepoints (Fig. 2). Of note, no dose-related trends were observed in myelosuppression or pancreatic events.

Ten patients (8.7%) experienced arterial occlusive events (AOEs) (Supplementary Table S6). Grade 1/2 angina pectoris was reported in 4 patients (3.5%) and resolved in all (1 requiring dose interruption and 2 requiring concomitant medication). Grade 3 events were reported in 5 patients (myocardial infarction, *n* = 2; myocardial ischemia, *n* = 1; coronary artery disease, *n* = 1; peripheral arterial occlusive disease and arterial bypass occlusion, *n* = 1). No grade 4 AOEs occurred. Of the 10 patients with AOEs, 8 had prior exposure to dasatinib, 6 to nilotinib, 5 to bosutinib, and 1 to ponatinib. Most had ≥1 baseline cardiovascular (CV) risk factor, including history of hyperlipidemia (*n* = 8), hypertension (*n* = 6), obesity (*n* = 4), and prior AOEs (*n* = 4); smoking status was not collected. No treatment discontinuations occurred due to AOEs; dose adjustments or interruptions occurred in 3 (2.6%) and 4 (3.5%) patients, respectively. One patient who had a myocardial ischemia event died due to cardiac arrest (unrelated to asciminib; see “Patients” and Supplementary Materials).

Seven patients (6.1%) had cardiac failure–related events (grade 3, *n* = 4 [3.5%]; grade 4, *n* = 1 [0.9%]), including 2 who also experienced AOEs (Supplementary Table S7); no treatment discontinuations occurred due to these events, and treatment interruption occurred in 3 patients (2.6%). Of the 7 patients, 5 had received 4 prior TKIs (previous ponatinib, *n* = 1); most had ≥1 baseline CV risk factor, including hyperlipidemia (*n* = 5), hypertension (*n* = 5), obesity (*n* = 3), and prior cardiac conditions (*n* = 4).

### Efficacy

**Fig. 3:**
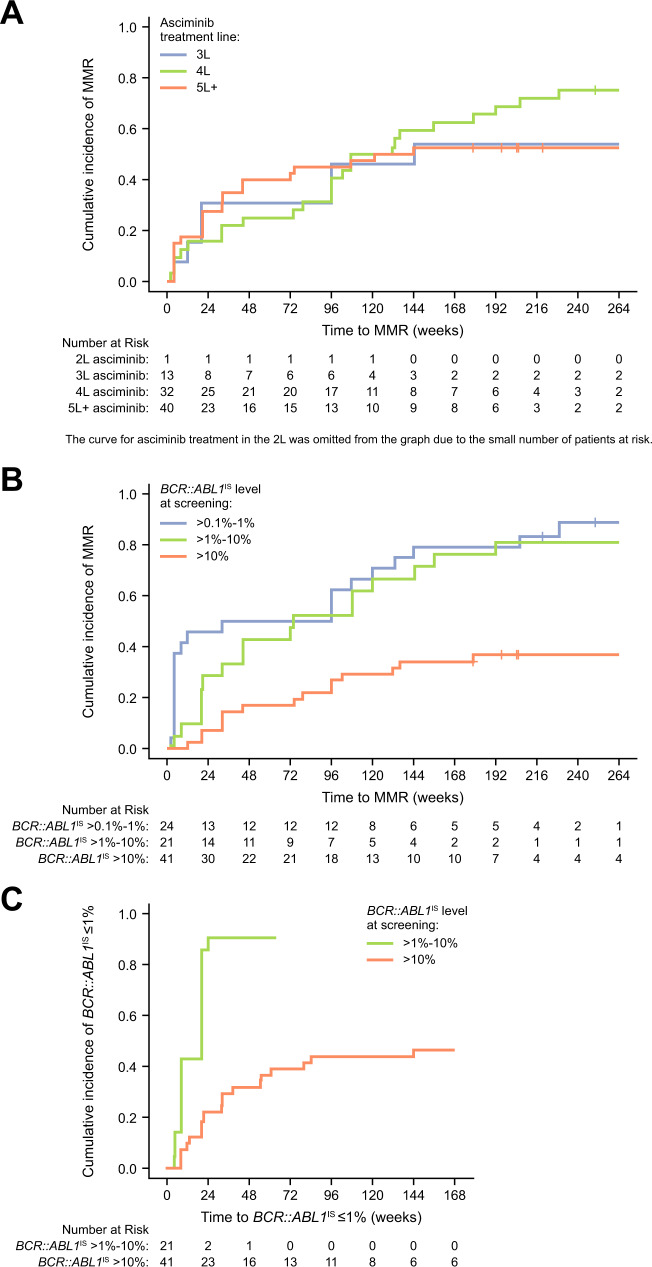
Cumulative rate of molecular response. Cumulative rate of **A** MMR by number of lines of prior TKI therapy, **B** MMR by *BCR::ABL1*^IS^ level at screening, and **C** *BCR::ABL1*^IS^ ≤ 1% by *BCR::ABL1*^IS^ level at screening. Treatment discontinuations for any reason were treated as competing events. *BCR::ABL1*^IS^ *BCR::ABL1* on the International Scale, L line of asciminib treatment (e.g., 3L indicates patients who were treated with asciminib in the third line), MMR major molecular response (*BCR::ABL1*^IS^ ≤ 0.1%), TKI tyrosine kinase inhibitor.

Of 115 patients, 9 had atypical or unknown transcripts (*BCR::ABL1*^IS^ could not be determined) and were excluded from all molecular response analyses. After excluding 20 additional patients who were in MMR at screening, 86 who were evaluable remained (*BCR::ABL1*^IS^ > 0.1% at screening) for analysis of cumulative MMR. Of these 86, 53 (61.6%) achieved MMR by data cutoff (Table 4 and Supplementary Fig. S5). Most responses were achieved by week 48; however, the cumulative MMR rate continued to increase over time, with the first MMR being attained by up to week 228 of treatment (median time to MMR based on time-to-event analysis, 132 weeks; 95% CI, 96–206 weeks). Cumulative MMR rates ranged from 52.5% to 75.0% in patients receiving asciminib in the third, fourth, or later lines (Fig. 3A). There was no obvious correlation between MMR rate and treatment line; similar trends of increasing response over time were observed. MMR rates were similar between patients with *BCR::ABL1*^IS^ > 0.1% to 1% (87.5%) and *BCR::ABL1*^IS^ > 1% to 10% at screening (81.0%) (Fig. 3B); half of the patients with *BCR::ABL1*^IS^ > 0.1% to 10% at screening achieved MMR by week 72, with additional responses reported at later timepoints. Of 41 patients with *BCR::ABL1*^IS^ > 10% at screening, 15 (36.6%) achieved MMR by data cutoff; approximately half (*n* = 8) achieved MMR by week 72.

**Table 4:** Cumulative incidence of molecular response by time point.

Time point, *n* (%)	MR^2^ (*n* = 62)^a^	MMR (*n* = 86)^a^	MR^4^ (*n* = 101)^a^	MR^4.5^ (*n* = 105)^a^
Overall (by the data cutoff)	38 (61.3)	53 (61.6)	34 (33.7)	32 (30.5)
By week 24	30 (48.4)	20 (23.3)	15 (14.9)	14 (13.3)
By week 48 (≈year 1)	33 (53.2)	28 (32.6)	19 (18.8)	19 (18.1)
By week 72	35 (56.5)	31 (36.0)	22 (21.8)	21 (20.0)
By week 96 (≈year 2)	37 (59.7)	37 (43.0)	23 (22.8)	22 (21.0)
By week 120	38 (61.3)	43 (50.0)	23 (22.8)	23 (21.9)
By week 144 (≈year 3)	38 (61.3)	48 (55.8)	26 (25.7)	25 (23.8)
By week 168	38 (61.3)	49 (57.0)	28 (27.7)	25 (23.8)
By week 192 (≈year 4)	38 (61.3)	51 (59.3)	28 (27.7)	27 (25.7)

*IS* International Scale, *MMR* major molecular response (*BCR::ABL1*^IS^ ≤ 0.1%), *MR*^*2*^ *BCR::ABL1*^IS^ ≤ 1%; *MR*^*4*^ *BCR::ABL1*^IS^ ≤ 0.01%; *MR*^*4.5*^ *BCR::ABL1*^IS^ ≤ 0.0032%.

^a^Patients with the corresponding molecular response level or atypical/unknown *BCR::ABL1* transcripts at screening were excluded from the analysis.

Of 53 patients who achieved MMR, 5 lost MMR by data cutoff, while 48 maintained MMR or achieved deeper responses. Of 20 patients who were in MMR at screening, the majority maintained or achieved deeper responses, and only 1 lost MMR by data cutoff. The Kaplan–Meier estimated rate of durable MMR at 96 weeks was 94% (95% CI, 86.4%–100.0%) among patients not in MMR at screening who achieved MMR at any time and 94.4% (95% CI, 84.4%–100%) among patients in MMR at screening. All, except 1 of the 6 patients who lost MMR, received asciminib in the fourth line or later (Supplementary Fig. S3). By data cutoff, 2 of these 6 patients had discontinued treatment after loss of MMR (1 due to AE and 1 due to lack of efficacy).

Sixty-two patients were evaluable (*BCR::ABL1*^IS^ > 1% at screening) in the analysis of cumulative *BCR::ABL1*^IS^ ≤ 1%. Thirty-eight (61.3%) achieved *BCR::ABL1*^IS^ ≤ 1% by data cutoff, with most achieving this response by week 24 (Table 4 and Supplementary Fig. S5). Of 21 patients with *BCR::ABL1*^IS^ > 1% to 10% at screening, nearly all (*n* = 19 [90.5%]) achieved *BCR::ABL1*^IS^ ≤ 1% by week 24 (Fig. 3C). Of 41 patients with *BCR::ABL1*^IS^ > 10% at screening, 19 (46.3%) achieved *BCR::ABL1*^IS^ ≤ 1% by data cutoff.

There were 101 and 105 patients who had *BCR::ABL1*^IS^ > 0.01% and >0.0032% at screening, respectively, and were thus evaluable for analyses of cumulative MR^4^ and MR^4.5^, respectively. Nearly one-third of these patients achieved DMR (MR^4^, *n* = 34 [33.7%]; MR^4.5^, *n* = 32 [30.5%]) by data cutoff, with most responses achieved by week 36 (Table 4 and Supplementary Fig. S5). The cumulative MR^4.5^ rate showed similar trends regardless of treatment line (Supplementary Fig. S3). Of 41 patients who had *BCR::ABL1*^IS^ > 10% at screening, 3 achieved MR^4.5^ by data cutoff (1 received third-line asciminib; 2 received fourth-line asciminib).

**Fig. 4:**
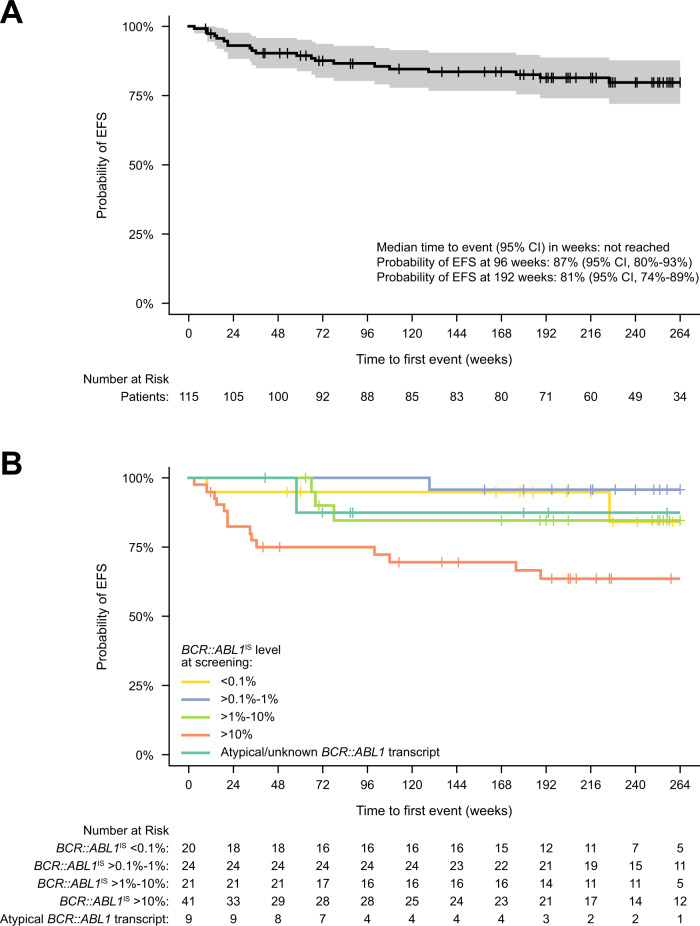
Event-free survival. **A** EFS for patients overall. **B** EFS by *BCR::ABL1*^IS^ at screening. EFS was estimated using the Kaplan–Meier method, with treatment discontinuation due to AEs, on-treatment progression to AP/BC, and on-treatment death for any reason considered as events. Survival data were not collected after patients discontinued the study. AE adverse event, AP accelerated phase, BC blast crisis, EFS event-free survival.

The Kaplan–Meier estimated EFS rate at 96 weeks was 87% (95% CI, 80%–93%), and median time to EFS was not reached (Fig. 4). Of 12 patients with *BCR::ABL1* mutations detected at screening, 6 achieved MMR by data cutoff (Supplementary Table S3). Four of these 12 (2 of whom achieved MMR) had additional treatment-emergent mutations (M244V, V289I, and myristoyl-pocket mutations G463S, V468F, and I502L) detected post screening; 3 of the 4 discontinued treatment (AE, *n* = 1; PD, *n* = 2). One patient with no mutations at screening had a newly emerged myristoyl-pocket mutation (G463D) detected post screening, did not achieve MMR, and discontinued treatment (physician decision/lack of efficacy). Details of these cases are reported in the Supplementary Materials.

## Discussion

Updated analysis of this phase 1 trial in heavily pretreated patients with CML-CP without T315I demonstrates the continued safety, tolerability, and substantial, durable efficacy of asciminib; with a median exposure of ≈4 years, most patients (69.6%) remained on asciminib. No new safety signals arose in this patient population. A significant proportion of patients achieved MMR or DMR, and very few lost MMR. The cumulative rate of MMR and DMR continued to increase with additional patients achieving responses even 3 years after initiation of treatment. Discontinuation due to PD (6.1%) and AEs (11.3%) occurred infrequently; 2 patients died on treatment, unrelated to asciminib. In studies of other TKIs (including nilotinib, dasatinib, bosutinib, and ponatinib) in heavily pretreated patients, 24%–62% remained on treatment [9, 10, 17, 19, 21, 22, 24, 34–37], including 24% to ≈56% in bosutinib studies [9, 24] and 33%–53% in ponatinib studies [10, 37].

The most frequently reported AEs grouped by common pathophysiological features were GI toxicity (72.2%), musculoskeletal pain (59.1%), hypersensitivity (mainly dermatologic events [44.3%]), and upper respiratory tract infection (41.7%); these events were generally mild and manageable. AEs (including hematologic) generally occurred early after treatment initiation, a pattern also observed in the phase 3 ASCEMBL trial, where most first-ever AEs (including hematologic) occurred in the first 6 months [30]. Myelosuppression overall was reported in one-third of patients (34.8%); these events mainly occurred early, particularly thrombocytopenia (25.2%), which was reported only in the first year. The short latency period suggests potent suppression of the leukemic clone with incomplete recovery and/or both disease- and therapy-related inhibition of nonleukemic hematopoiesis [38].

Pancreatic toxicity, including pancreatic enzyme elevations, is a broad safety concern for patients with CML receiving TKIs [2, 11, 39]. The pancreas was identified as a target organ of asciminib toxicity in dogs (but not rats and monkeys) [40]. However, the risk factors and mechanism of pancreatic toxicity with TKIs targeting ABL, including asciminib, are unknown. In this long-term clinical setting, events were mainly asymptomatic pancreatic enzyme elevations (40%) and generally manageable with dose adjustments, leading to discontinuation in only 3.5% of patients. Clinical pancreatitis was reported in 8 patients (7.0%) and led to discontinuation in 2 (1.7%). Of note, no cases of pancreatitis were reported in ASCEMBL, and grade ≥3 increased lipase occurred in only 3.8% of patients receiving asciminib [28]. Regular monitoring of pancreatic function is recommended during asciminib treatment, with dose interruption or reduction as needed.

CV toxicity is observed with all ABL kinase inhibitors, including ponatinib, and warrants a personalized approach for optimal CV risk management in patients with CML [41, 42]. Due to multiple confounding factors, including heavy pretreatment with TKIs that potentially exacerbated or led to the development of baseline CV risk factors in patients experiencing AOEs (8.7%) or cardiac failure events (6.1%) in this analysis, the causal or contributory role of asciminib remains uncertain. Importantly, data on long-term exposure from this analysis are reassuring; no increase in the frequency or severity of AOEs with longer asciminib exposure was observed. With longer follow-up, the safety profile of asciminib remained unchanged compared with previously published data [29], raising no new concerns.

MMR is a well-established treatment goal [2] and is associated with improved outcomes, including OS and PFS [43–46]. However, achieving MMR in third or later lines of therapy may be difficult; major treatment goals for these patients include preventing PD and achieving and maintaining other protective response thresholds, including *BCR::ABL1*^IS^ ≤ 1% [1, 2]. Many patients in this trial (61.3%) achieved *BCR::ABL1*^IS^ ≤ 1% by data cutoff, including 53.2% by 48 weeks (≈12 months). Although other trials have reported similar response in patients with CML-CP without T315I after ≥2 prior TKIs, responses appear to be achieved earlier with asciminib; in ASCEMBL, 50.8% of patients receiving asciminib had this response by 48 weeks (≈12 months) [47], whereas, in the OPTIC trial, 46.2% of patients receiving ponatinib achieved this response by 36 months [48]. In the PACE trial, 49% of patients with CML without T315I after ≥1 prior TKI achieved a correlate of *BCR::ABL1*^IS^ ≤ 1% by 57 months [2, 10].

In this updated analysis, the cumulative MMR rate continued to increase with longer asciminib treatment duration regardless of line of therapy or baseline disease characteristics, with two-thirds of patients (61.6%) achieving MMR by data cutoff; rates ranged from 52.5% to 75.0% by treatment line, confirming the benefit of asciminib in this heavily pretreated population for whom options are very limited. These data compare favorably with those in other later-line TKI studies [10, 13, 19, 20, 22, 34, 35, 49]. Acknowledging potentially variable response levels prior to change in therapy among these studies, MMR rates were 15% in patients with or without T315I receiving third- and fourth-line bosutinib (median follow-up, 28.5 months) [49] and 35% in patients without T315I in the PACE 5-year analysis of third-line or later ponatinib [10].

Compared with lesser depth of response, DMR is associated with improved outcomes, including better OS, EFS, and failure-free survival and reduced risk of progression to AP/BC [50–52] and is a required level of response for treatment-free remission eligibility [1, 2]. Approximately one-third (33.7%) of evaluable patients in this analysis achieved MR^4^ with asciminib; this appears favorable compared with 26% in a 5-year analysis of patients without T315I treated with ponatinib, with the caveat that response levels prior to treatment may have differed from those in the current study [10]. Of 41 patients receiving asciminib with *BCR::ABL1*^IS^ > 10% at screening (a highly refractory population), 46.3% achieved *BCR::ABL1*^IS^ ≤ 1%, 36.6% achieved MMR, and 7.3% (*n* = 3) achieved MR^4.5^.

This longer-term follow-up demonstrates the durability of response achieved with asciminib; only 5 of 53 patients who achieved MMR lost this response (2 of 5 remain in *BCR::ABL1*^IS^ ≤ 1%). This durability, with median time to MMR of 132 weeks with asciminib, appears favorable compared with that of ponatinib in the PACE 5-year analysis, in which ≈41% of patients without T315I who achieved MMR lost this response and median time to MMR was not reached [10].

Newly emerged *BCR::ABL1* mutations were detected in 5 patients (M244V, V289I, and myristoyl-pocket mutations G463D, G463S, V468F, and I502L). Of 12 patients with mutations detected at screening (E255K, F317L, G250E, L248V, V299L, M244V, Y253H), 6 achieved MMR; however, at this time, data are too limited to draw conclusions on the impact of mutations on the efficacy of asciminib.

In conclusion, asciminib, with its novel mechanism specifically targeting the ABL myristoyl pocket, is safe, well tolerated, and provides durable longer-term responses, including DMR in heavily pretreated patients with CML-CP without T315I. With a median treatment duration of ≈4 years, more than two-thirds of patients remained on therapy. These extended safety and response data from the phase 1 study complement results from ASCEMBL, which demonstrated superior efficacy and favorable safety with asciminib compared with bosutinib [28, 30]. Expanded clinical investigation of asciminib is warranted, including in patients with newly diagnosed CML-CP. A phase 3 trial (NCT04971226) is enrolling patients with CML-CP to evaluate asciminib versus all other approved first-line TKIs (imatinib, nilotinib, dasatinib, and bosutinib). This trial, along with other planned and ongoing studies and routine pharmacovigilance activities, will further characterize the efficacy and safety profile of asciminib, collect additional mutation data, and define optimal use for patients with CML.

## Supplementary information

Supplementary Material

Supplementary Table S3

Supplementary Table S5

Supplementary Table S6

Supplementary Table S7

Supplementary Table S8

Supplementary Table S9

## Data Availability

Novartis is committed to sharing access to patient-level data and supporting clinical documents from eligible studies with qualified external researchers. These requests will be reviewed and approved by an independent review panel based on scientific merit. All data provided will be anonymized to respect the privacy of patients who have participated in the trial, consistent with applicable laws and regulations. This trial data availability follows the criteria and process described at https://www.clinicalstudydatarequest.com/.
