# Supplementary material for: Asciminib monotherapy in patients with CML-CP without BCR::ABL1 T315I mutations treated with at least two prior TKIs: 4-year phase 1 safety and efficacy results
Source: Leukemia. 2023 Mar 22;37(5):1048–59. doi: 10.1038/s41375-023-01860-w (PMC10169635; doi:10.1038/s41375-023-01860-w)
Supplement: Supplementary file 1 — Supplementary Material [file 41375_2023_1860_MOESM1_ESM.pdf]

Asciminib Monotherapy in Patients With CML-CP Without *BCR::ABL1* T315I Mutations Treated With at Least 2 Prior TKIs: 4-Year Phase 1 Safety and Efficacy Results

SUPPLEMENTAL MATERIALS

SUPPLEMENTAL METHODS

Study Design

Enrolled patients were allocated to 1 of 5 study arms (**Supplemental Figures S1 and S2**). In study arm 1, patients with chronic myeloid leukemia (CML) in chronic phase (CP) or accelerated phase (AP) received asciminib monotherapy at different doses and on 2 administration schedules (once daily and twice daily). In study arm 2, patients with CML in CP or AP received asciminib combined with nilotinib. In study arm 3, patients with CML in CP or AP received asciminib combined with imatinib. In study arm 4, patients with CML in CP or AP received asciminib combined with dasatinib. In study arm 5, patients with CML in blast phase (BP) or with Philadelphia chromosome–positive (Ph+) acute lymphoblastic leukemia (ALL) received asciminib monotherapy.

Each study arm began with a dose-escalation part. Following determination of the maximum tolerated dose or recommended dose for expansion, safety and tolerability were further evaluated in a dose-expansion part. Patients continued asciminib until progressive disease (PD), unacceptable toxicity, investigator decision, or withdrawal of consent. Patients were not allowed to attempt treatment-free remission on study.

Study Assessments

Molecular responses were assessed at screening, monthly until cycle 3 (or cycle 4 for patients enrolled in dose-expansion cohorts and upon implementation of protocol amendment 6), and then every 3 months or as clinically indicated. Each cycle was 28 days. Mutational analyses were to be performed in patients at screening, for unconfirmed loss of response, and as needed.

Methods for Defining Dose-Limiting Toxicities

Dose-limiting toxicities (DLTs), which were assessed as part of the primary analysis, were defined as adverse events (AEs) or abnormal laboratory values that investigators deemed unrelated to PD, intercurrent illness, or concomitant medication use; occurred within the first 28 days (cycle 1) of study treatment; and met any of the criteria in **Supplemental Table S8.** The final analysis of DLT data is reported here.

Statistical Analyses

AEs were reported using numbers and percentages of patients. A patient with multiple grades for an AE was reported under the maximum grade for that AE. AEs were reported as grouped preferred terms of similar events considered clinically meaningful, where applicable. The preferred term “hyperlipasemia” was consolidated into the preferred term “increased lipase” because these are identical events.

Clinically important safety information was identified and presented by grouping AEs that are of scientific and medical interest specifically for asciminib and/or are related to class risks of other TKIs. Safety information was also based on the totality of evidence from preclinical and clinical studies and the potential impact on the benefit-risk profile of asciminib. Within each specified clinically important safety information category, numbers and percentages of patients with ≥1 AE were summarized.

The time to and duration of first major molecular response (MMR; *BCR::ABL1* ≤0.1% on the International Scale [IS]) were estimated using the Kaplan-Meier method. Time to MMR was defined as the time between the study start date and the date when *BCR::ABL1*^{IS} ≤0.1% was first observed. Duration of MMR was defined as the time between the date when *BCR::ABL1*^{IS} ≤0.1% was first observed and the date of confirmed loss of MMR. Patients were censored at the date of their last *BCR::ABL1* assessment. Loss of MMR was defined as *BCR::ABL1*^{IS} >0.1% at any time point after achievement of MMR, in association with a ≥5-fold increase in *BCR::ABL1*^{IS} from the lowest value achieved up to that time point, and was confirmed by a subsequent sample analysis. Subsequent sample analysis was not required for patients discontinuing treatment prematurely due to CML-related death or PD.

A second analysis of estimated event-free survival (EFS) was conducted, with treatment discontinuation due to AEs, on-treatment progression to AP/blast crisis (BC), on-treatment death

for any reason, *BCR::ABL1*^{IS} >10% at 6 months, and *BCR::ABL1*^{IS} >1% at ≥12 months considered events. Survival data were not collected after patients discontinued the study.

Analyses of DLTs and pharmacokinetics (PK) are reported for patients with CML in CP or AP regardless of T315I status at screening. All other analyses in this report are in patients with CML-CP without T315I.

PK Methods

PK data were analyzed from patients with CML in CP or AP, regardless of T315I status at screening, who received asciminib monotherapy. Analyses were conducted with the PK analysis set, which consisted of all patients who had ≥1 blood sample providing an evaluable full PK profile (cycle 1, day 1; cycle 1, day 15; or cycle 2, day 1). The final analysis of PK data is reported here.

SUPPLEMENTAL RESULTS

DLTs

As of the data cutoff, 132 patients with CML in CP or AP with or without T315I who were enrolled to receive asciminib monotherapy across the treatment cohorts (10 mg twice daily, n=1; 20 mg twice daily, n=5; 40 mg twice daily, n=12; 80 mg twice daily, n=12; 150 mg twice daily, n=13; 160 mg twice daily, n=11; 200 mg twice daily, n=26; 80 mg once daily, n=18; 120 mg once

daily, n=22; and 200 mg once daily, n=12) in the dose-escalation part of the study were eligible for the dose-determining analysis set.

DLTs were reported in 8 of these 132 patients (6.1%) during the first cycle of treatment and consisted of grade 3 lipase increased (n=1; 40 mg twice daily), grade 2 arthralgia and myalgia (n=1; 80 mg twice daily), grade 3 acute coronary syndrome (n=1; 150 mg twice daily), grade 4 thrombocytopenia (n=1; 160 mg twice daily), grade 3 bronchospasm (n=1; 200 mg twice daily), grade 3 pancreatitis (n=2; 200 mg once daily), and grade 3 lipase increased (n=1; 200 mg once daily). For asciminib monotherapy in patients with CML-CP/AP without T315I, 40 mg twice daily was confirmed as the recommended phase 2 dose. This dose is currently being evaluated in the phase 3 ASCEMBL clinical trial (NCT03106779) in patients with CML-CP without T315I (1).

On-Treatment Deaths (On Treatment or ≤30 Days After Last Study Drug Dose)

A 65-year-old female patient who was enrolled in the study after 4 prior tyrosine kinase inhibitors (TKIs) was treated with asciminib for >3 years until death (1 day after last asciminib dose) due to cardiac arrest that was considered not related to study treatment; this event was not considered related to QT prolongation, and no relevant changes in QT interval adjusted according to Fredericia were observed during the long treatment duration. This patient had hypercholesterolemia and carotid artery

stenosis and had comorbidities, including systemic scleroderma, hypertension, acute kidney injury, and ischemic heart disease, that were present at the time of the event and contributed to the fatal cardiac arrest.

A 61-year-old male patient who was enrolled in the study after 2 prior TKIs had multiple comorbidities that evolved during the study, including liver enzymes elevation, lymphopenia, hyperuricemia, multiple events of kidney failure, hematuria, sepsis, liver disorders, and anemia. The patient had a history of bladder cancer and urostomy. After ≈1 year of treatment with asciminib, the patient died (2 days after last asciminib dose) due to worsening of general physical condition, including multiple organ failure and malnutrition. None of the events leading to death were reported as being possibly related to study treatment.

Off-Treatment Deaths (>30 Days After Last Study Drug Dose)

A 70-year-old male patient who was enrolled in the study after 3 prior TKIs had multiple cardiovascular comorbidities, including hypertension, diabetes mellitus II, coronary artery disease with 2 stents, hypertriglyceridemia, and Sjogren syndrome. The patient experienced multiple episodes of grade 3 peripheral arterial occlusive disease, which the investigator did not report as related to study treatment; the last episode did not resolve and required prolonged treatment interruption. The patient later died due to pneumonia aspiration 65 days after the last dose of asciminib before treatment could be restarted.

One patient discontinued study treatment on day 133 due to PD to CML BC (based on bone marrow aspirate). The patient started postantineoplastic therapy with daunorubicin and cytarabine and died 2 months later due to the underlying disease.

Clinically Important Safety Information

The vast majority of AEs related to hepatotoxicity were asymptomatic enzyme elevations; 4 patients had mild clinical events, and 1 experienced a grade 3 event of liver disorder (only 1 patient who died due to worsening of general physical condition had a grade 3 liver disorder that was serious and occurred in the context of concurrent renal failure and abdominal sepsis due to *Escherichia coli* [see *On-Treatment Deaths* and *Off-Treatment Deaths* in the **Supplementary Materials** for additional details]).

Four patients had grade 3 hypersensitivity events (bronchospasm, n=2 [1.7%]; circulatory collapse, n=1 [0.9%]; and dermatitis bullous, n=1 [0.9%]). In one of the patients who experienced grade 3 bronchospasm, this was a DLT event that led to treatment discontinuation, along with cyanosis (grade 3), and rash (grade 1). The grade 3 circulatory collapse occurred due to underlying cardiac etiology and was not related to a hypersensitivity reaction (one of the etiologies of circulatory collapse is severe anaphylaxis, and circulatory collapse is thus grouped with the clinically important safety information category of hypersensitivity; in this case, the etiology was underlying cardiac issues).

With regard to QT interval prolongation, clinically meaningful changes in the electrocardiogram interval were not observed; only 1 patient had a new QT interval adjusted according to Fredericia of >500 ms, which was not associated with cardiac symptoms.

Mutation Data

Of 12 patients with mutations detected at screening (**Table 1; Supplemental Table S3**), 6 achieved MMR at any time point while on asciminib treatment. Newly emergent mutations were detected post screening in 4 of the 12 patients who had mutations detected at screening, 3 of whom discontinued treatment (**Supplemental Table S3; Supplemental Figure S6**). One patient with E255K at screening achieved MMR during treatment, acquired a newly emergent G463S myristoyl pocket mutation post screening, and discontinued due to an AE of grade 3 pancreatitis. One patient with F317L at screening achieved MMR during treatment, acquired a newly emergent V289I mutation post screening, and remained on study at the data cutoff. One patient with G250E, L248V, and V299L at screening did not achieve MMR during treatment, had a newly emergent M244V mutation post screening, and discontinued due to progressive disease. One patient with V299L at screening did not achieve MMR during treatment, acquired newly emergent I502L and V468F myristoyl pocket mutations post screening, and discontinued due to progressive disease. One patient who did not have mutations

detected at screening and did not achieve MMR acquired a newly emergent G463D myristoyl pocket mutation post screening and discontinued due to physician decision (lack of efficacy).

EFS

An analysis of EFS was conducted, with treatment discontinuation due to AEs, on-treatment progression to AP/BC, on-treatment death for any reason, *BCR::ABL1*^{IS} >10% at 6 months, and *BCR::ABL1*^{IS} >1% at 12 months or later considered as events (**Supplemental Figure S7**). In this analysis, the Kaplan-Meier estimated EFS rate at 96 weeks was 67% (95% CI, 58%-76%), and median time to EFS was not reached.

PK Results

As of the data cutoff, 198 patients with CML in CP or AP with or without T315I who received asciminib monotherapy across the treatment cohorts were eligible for the PK analysis set. PK results in patients in the 40-mg twice-daily (n=32), 80-mg once-daily (n=18), and 200-mg twice-daily (n=62) dose groups are reported in **Supplemental Table S9.** In a prior publication, PK data for cycle 1, day 1 were reported for 31 patients at the 40-mg twice-daily dose level; 15 patients for the 80-mg once-daily dose level; and 8 patients for the 200-mg twice-daily dose level (2).

References

1. Rea D, Mauro MJ, Boquimpani C, Minami Y, Lomaia E, Voloshin S, et al. A phase 3, open-label, randomized study of asciminib, a STAMP inhibitor, vs bosutinib in CML after 2 or more prior TKIs. Blood. 2021;138(21):2031–41.

2. Hughes TP, Mauro MJ, Cortes JE, Minami H, Rea D, DeAngelo DJ, et al. Asciminib in chronic myeloid leukemia after abl kinase inhibitor failure. N Engl J Med. 2019;381(24):2315–26.

Supplemental Tables

Supplemental Table S1. Number of patients by starting asciminib dose[a]

**Asciminib starting dose**	**n**
All starting doses	115
10 mg twice daily	1
20 mg twice daily	13
40 mg twice daily	30
80 mg twice daily	8
150 mg twice daily	5
160 mg twice daily	3
200 mg twice daily	10
80 mg once daily	17
120 mg once daily	17
200 mg once daily	11

[a] The indicated asciminib doses were at the start of treatment; patients could have had dose adjustments during the course of treatment.

Supplemental Table S2. Patient race and ethnicity

**Race and ethnicity, n (%)**	**Patients (N=115)**
Race	
White	82 (71.3)
Asian	18 (15.7)
Black or African American	3 (2.6)
Other/unknown	12 (10.4)
Ethnicity	
East Asian	16 (13.9)
Hispanic or Latino	7 (6.1)
Southeast Asian	1 (0.9)
Other	57 (49.6)
Not reported/unknown	34 (29.6)

Supplemental Table S4. Overview of safety results

AEs, n (%)[a]	Patients (N=115)
All AEs	115 (100)
Grade ≥3 AEs	83 (72.2)
Grade 5 AEs	2 (1.7)
AEs leading to discontinuation	13 (11.3)
Increased lipase	4 (3.5)
Increased amylase[b]	2 (1.7)
Pancreatitis[c]	2 (1.7)
Thrombocytopenia[d]	2 (1.7)
Acute kidney injury[e]	1 (0.9)
Bronchospasm[f]	1 (0.9)
Cardiac arrest[e]	1 (0.9)
Cyanosis[f]	1 (0.9)
General physical condition abnormal	1 (0.9)
Leukocytosis	1 (0.9)
Rash[f]	1 (0.9)
Thrombocytosis	1 (0.9)
AEs leading to dose adjustment/interruption	69 (60.0)
AEs requiring additional therapy	105 (91.3)

AE, adverse event; PT, preferred term.

[a] Patients with multiple grades of severity for an AE were only counted under the maximum grade.

[b] The 2 patients who discontinued due to increased amylase also discontinued due to increased lipase.

[c] Includes PTs pancreatitis and pancreatitis acute.

[d] Includes preferred terms thrombocytopenia and platelet count decreased.

[e] Occurred in the same patient.

[f] Occurred in the same patient.

Supplemental Figures

Supplemental Figure S1. Study design

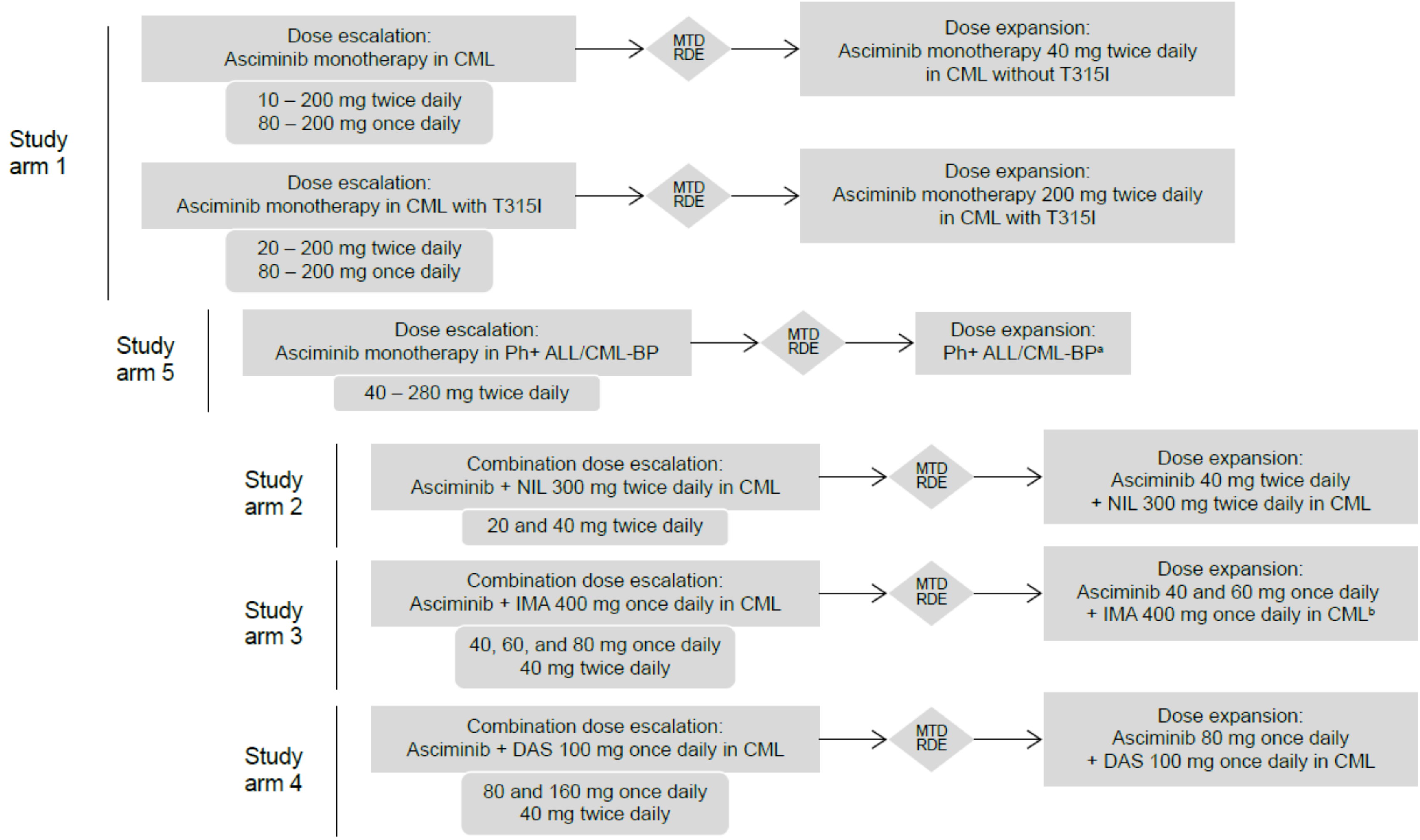

From Hughes TP, et al. *N Engl J Med*. 2019;381(24):2315–2326. Copyright © 2019 Massachusetts Medical Society. Reprinted with permission from Massachusetts Medical Society.

ALL, acute lymphoblastic leukemia; BP, blast phase; CML, chronic myeloid leukemia; DAS, dasatinib; IMA, imatinib; MTD, maximum tolerated dose; NIL, nilotinib; Ph+, Philadelphia chromosome positive; RDE, recommended dose for expansion.

[a] The RDE has not been determined, and no dose expansion cohort has been opened.

[b] Dose expansion of asciminib in combination with imatinib is being assessed in a separate phase 2 study (NCT03578367).

Supplemental Figure S2. Patient disposition for overall study[a]

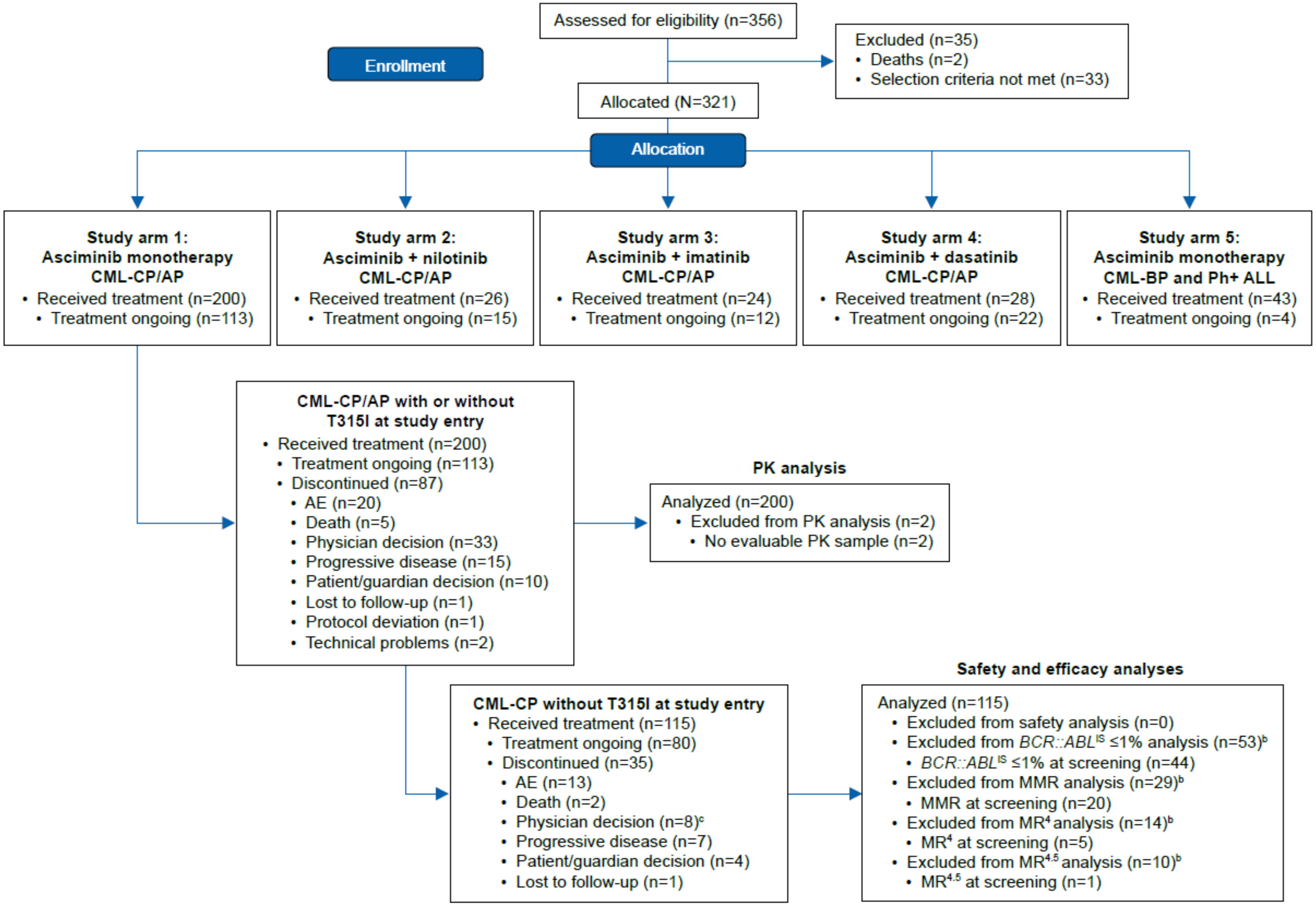

AE, adverse event; ALL, acute lymphoblastic leukemia; AP, accelerated phase; BP, blast phase; CML, chronic myeloid leukemia; CP, chronic phase; IS, International Scale; MMR, major molecular response (*BCR::ABL1* ≤0.1% on the IS); MR^{4}, *BCR::ABL1*^{IS} ≤0.01%; $MR^{4.5}$, *BCR::ABL1*^{IS} ≤0.0032%; Ph+, Philadelphia chromosome positive; PK, pharmacokinetics.

[a] Patient disposition as of the data cutoff date of January 6, 2021.

[b] Nine patients were excluded for having atypical/unknown *BCR::ABL1* transcripts at screening.

[c] Discontinuations were mainly due to lack of efficacy.

Supplemental Figure S3. Duration of asciminib treatment by line of therapy[a]

A. Patients who received third-line asciminib and had prior TKI duration available

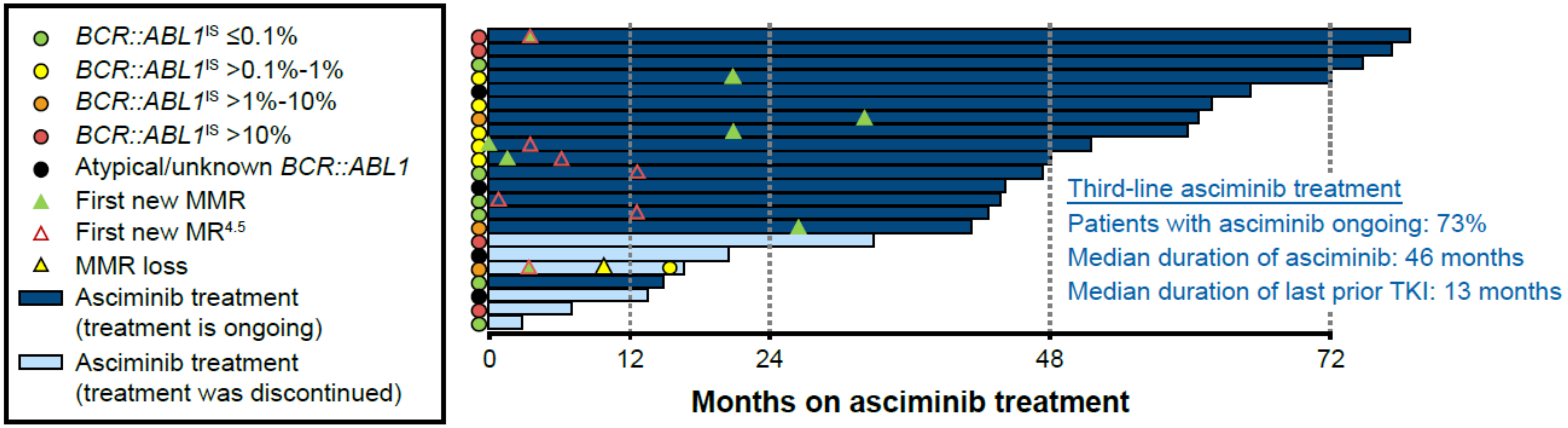

B. Patients who received fourth-line asciminib and had prior TKI duration available

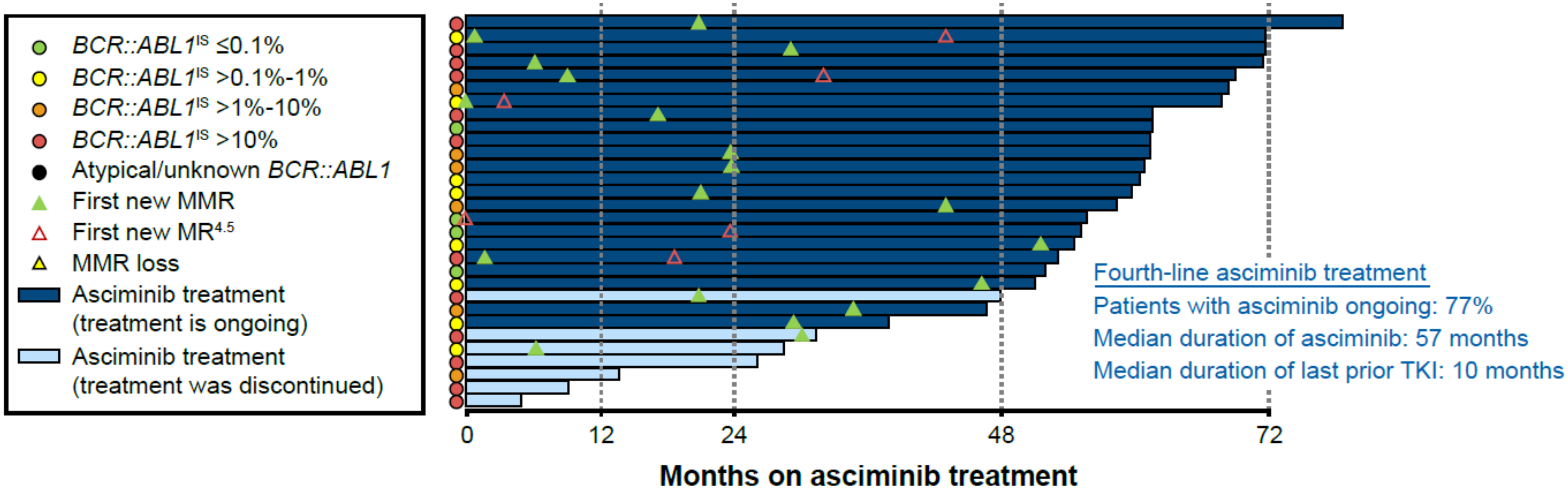

C. Patients who received fifth- or later-line asciminib and had prior TKI duration available

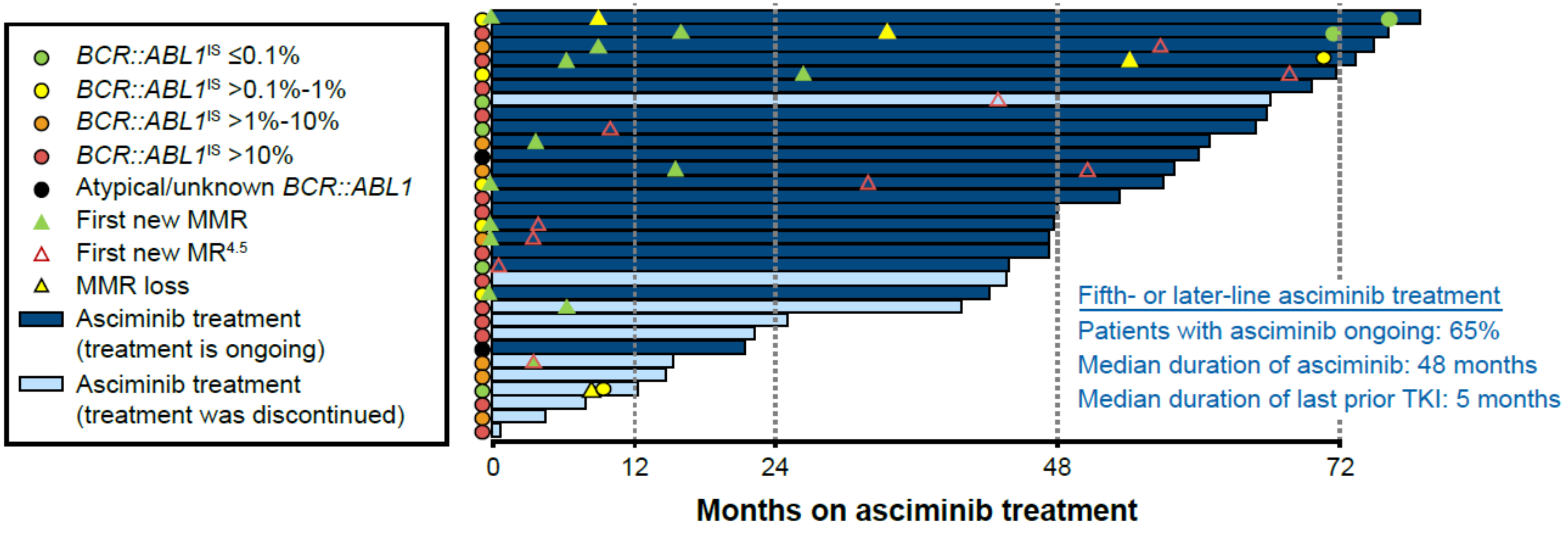

D. Patients for whom duration of last prior TKI was not available

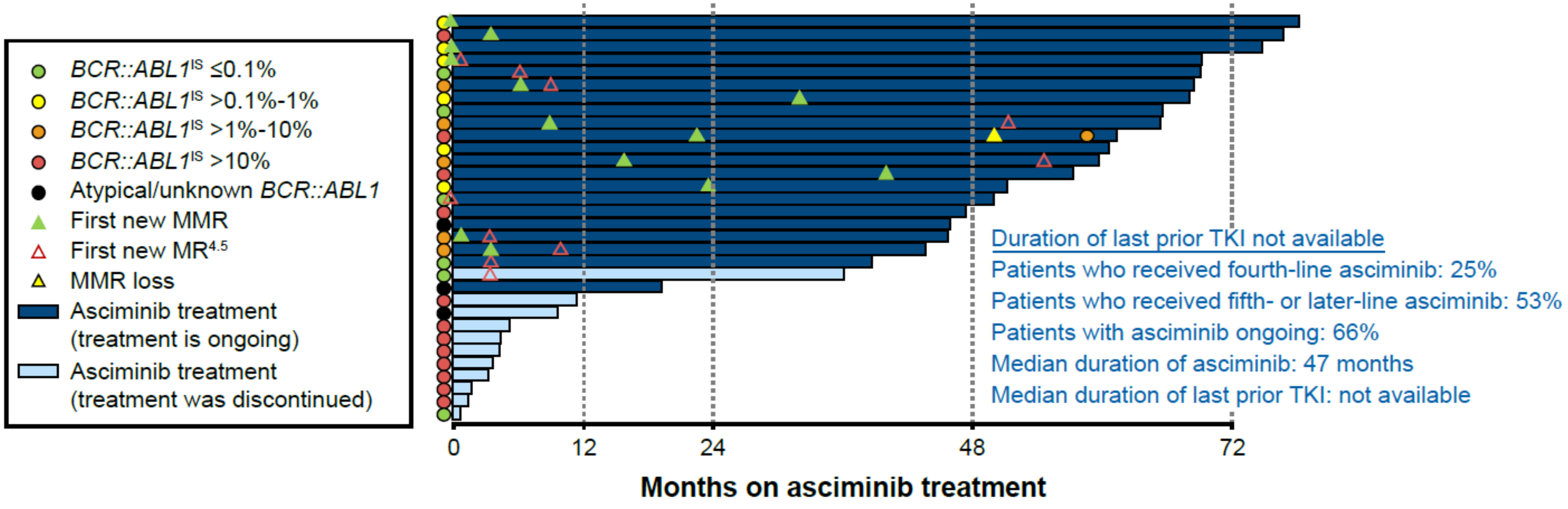

BCR::ABL1^{IS}, *BCR::ABL1* on the IS; IS, International Scale; MMR, major molecular response (*BCR::ABL1*^{IS} ≤0.1%); MR$^{4.5}$, *BCR::ABL1*^{IS} ≤0.0032%; TKI, tyrosine kinase inhibitor.

[a] Each bar corresponds to the duration of asciminib therapy for an individual patient. Dark bars indicate patients for whom asciminib treatment was ongoing at the data cutoff, and light bars indicate patients who had discontinued treatment by the data cutoff. Two patients who received asciminib in the second line were included in the group of patients who received asciminib in the third line. Median duration of asciminib treatment and the most recent prior TKI are indicated on the right of each figure panel. The duration of treatment with the most recent prior TKI could not be calculated for 32 patients (“Other” group). Colored circles on the left of each bar indicate *BCR::ABL1*^{IS} level at screening. Colored circles within bars indicate the last available *BCR::ABL1*^{IS} level for patients who lost MMR. Colored triangles within bars indicate first achievement of MMR or MR$^{4.5}$ for patients not in MMR or MR$^{4.5}$, respectively, at screening and first confirmed loss of MMR for any patient (excluding those with atypical/unknown transcripts). See figure legend for further information.

Supplemental Figure S4. Lipase and amylase levels over time in patients who experienced pancreatitis

A Patient 1

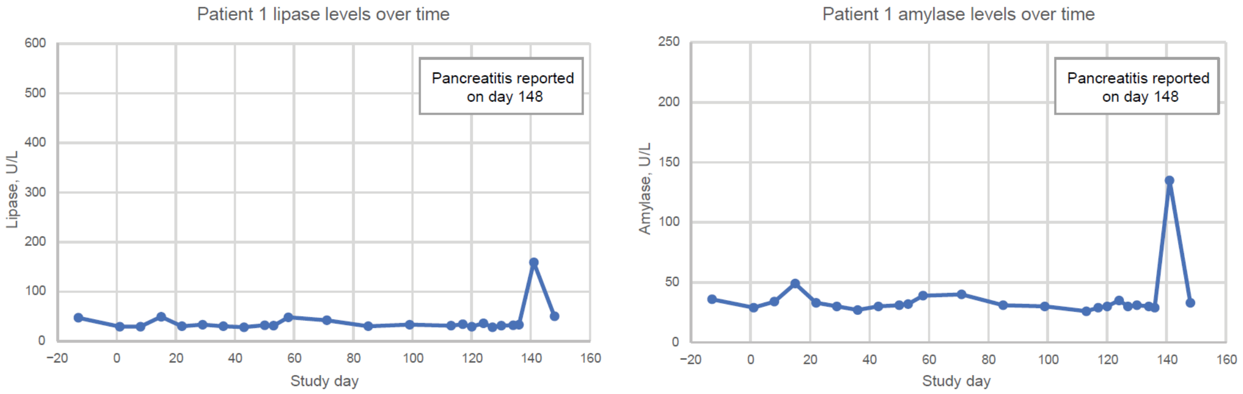

B Patient 2

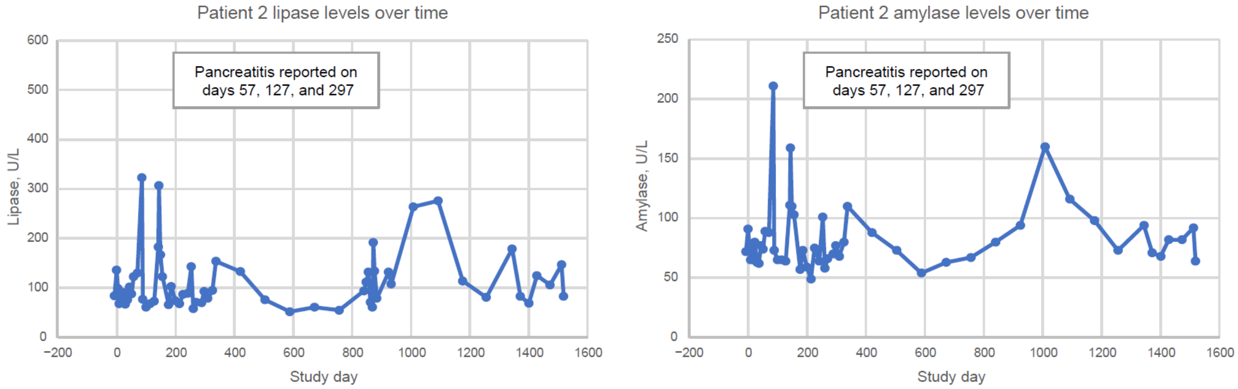

C Patient 3

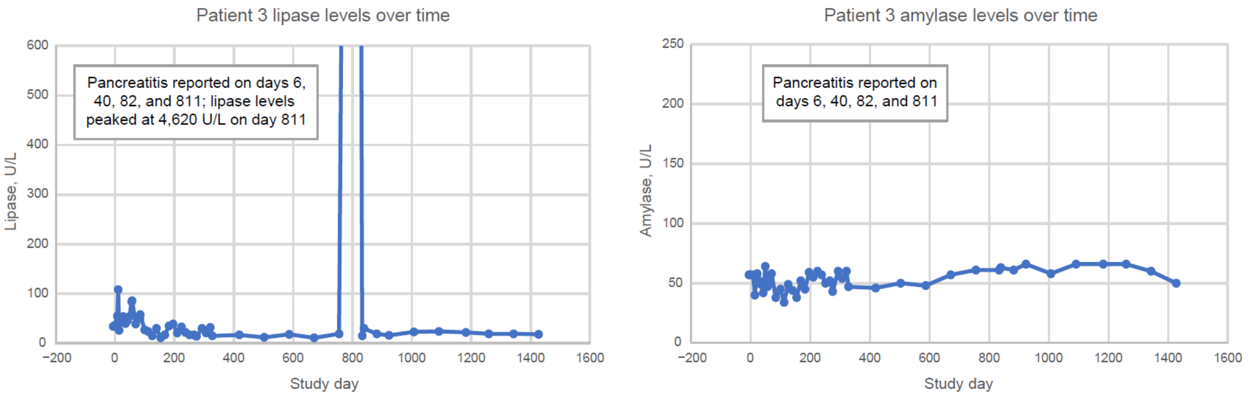

D Patient 4

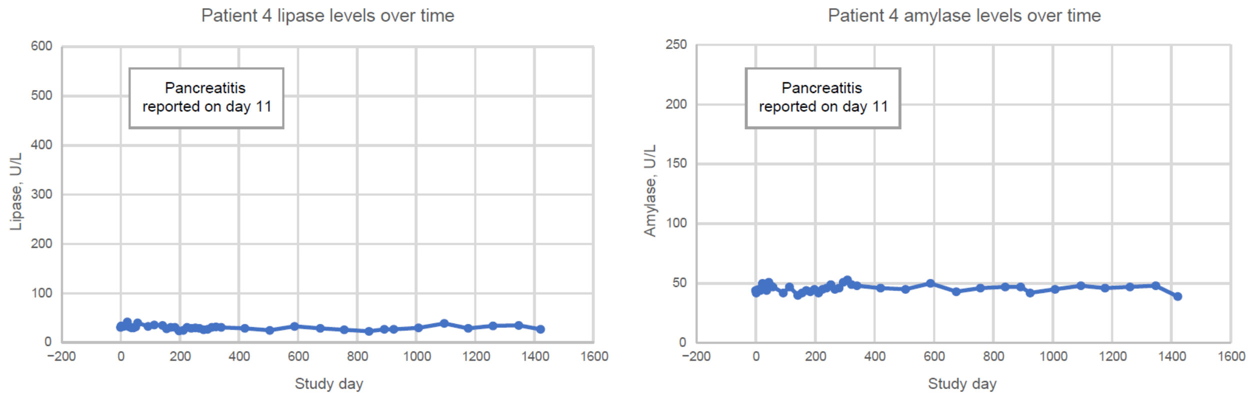

E Patient 5

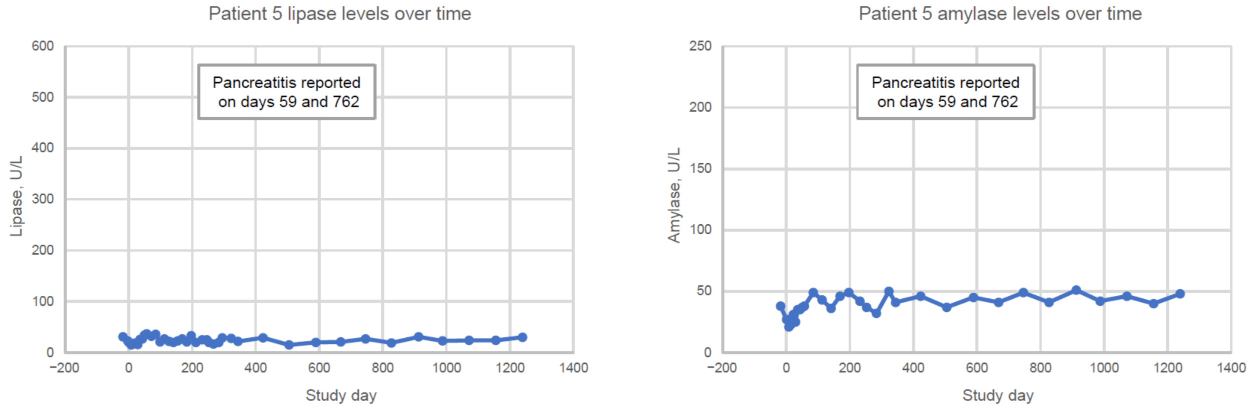

F Patient 6

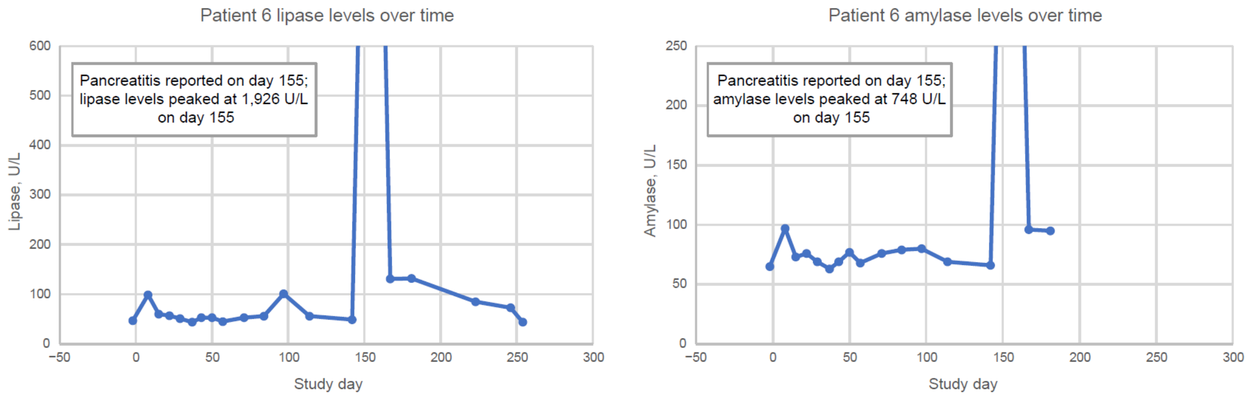

G Patient 7

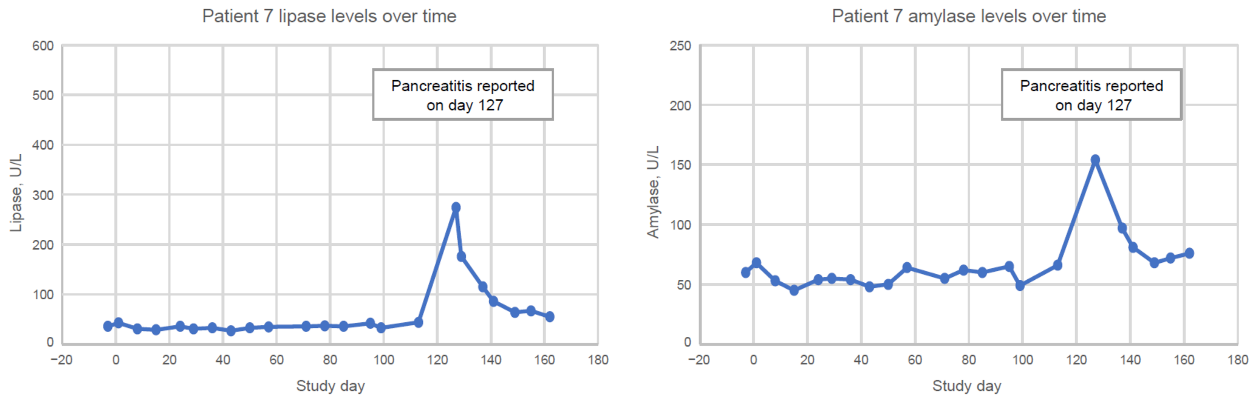

H Patient 8

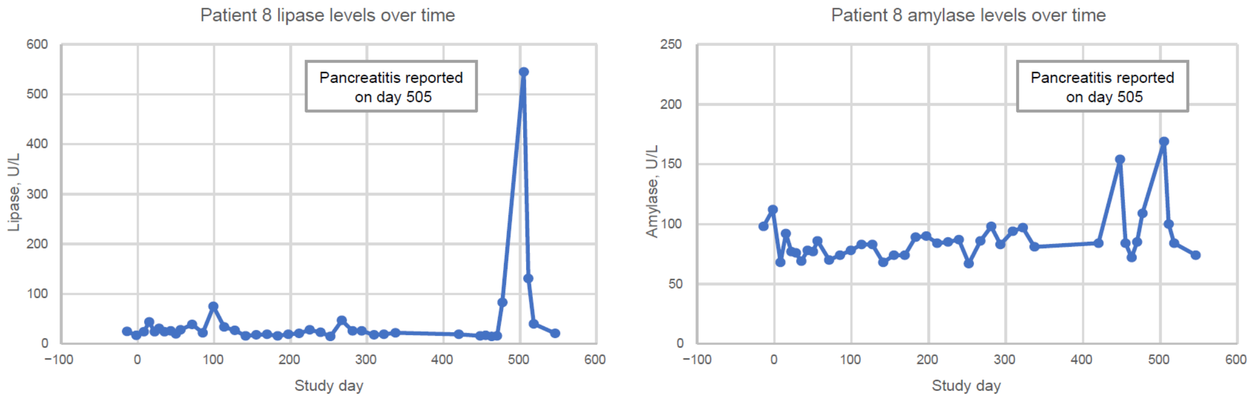

Supplemental Figure S5. Cumulative incidence of molecular response by time point[a]

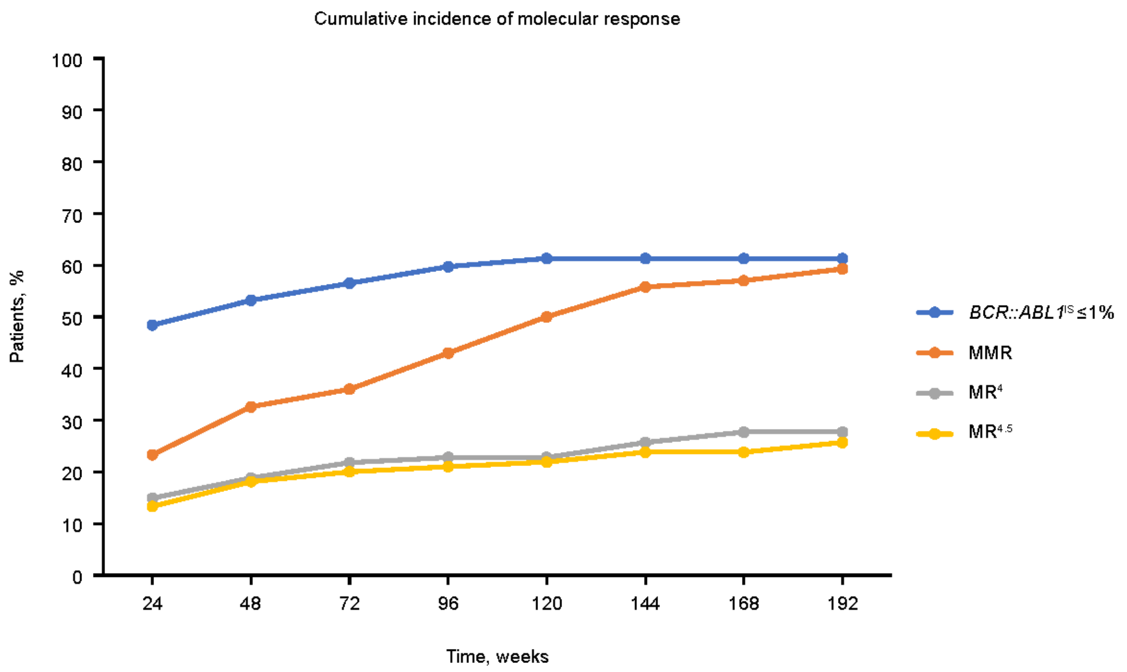

IS, International Scale; MMR, major molecular response (*BCR::ABL1*^{IS} ≤0.1%); MR^{4}, *BCR::ABL1*^{IS} ≤0.01%; MR$^{4.5}$, *BCR::ABL1*^{IS} ≤0.0032%.

[a] See **Table 4** for numbers of patients with these molecular response levels at each time point and numbers of evaluable patients. Patients with the corresponding molecular response level or atypical/unknown *BCR::ABL1* transcripts at screening were excluded from the analysis.

Supplemental Figure S6. Patients with mutations newly detected post screening[a]

A Patient 1 (I502L and V468F newly detected post screening)

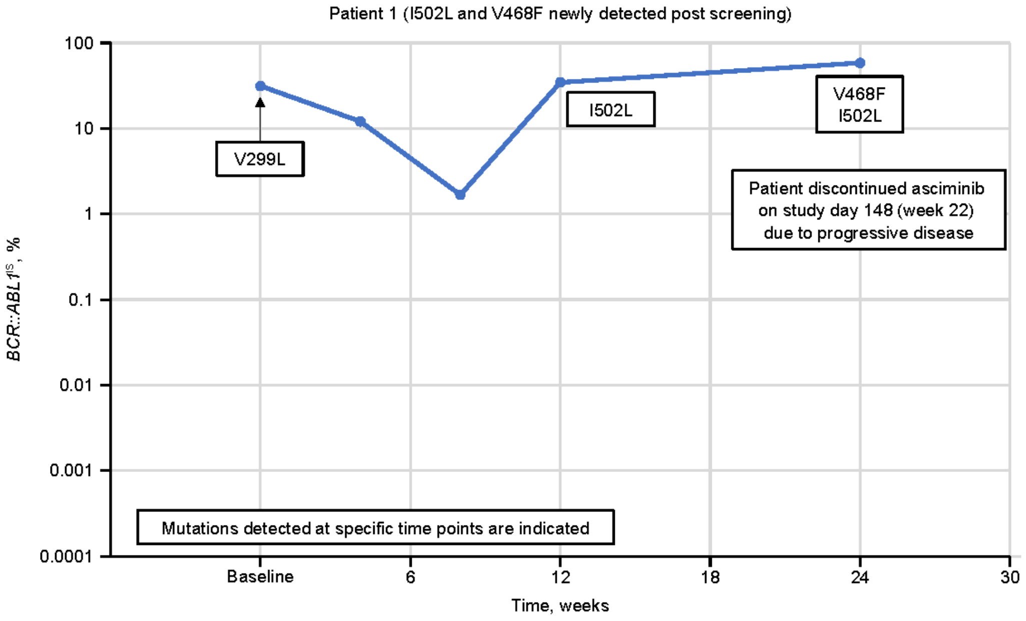

B Patient 8 (G463S newly detected post screening)

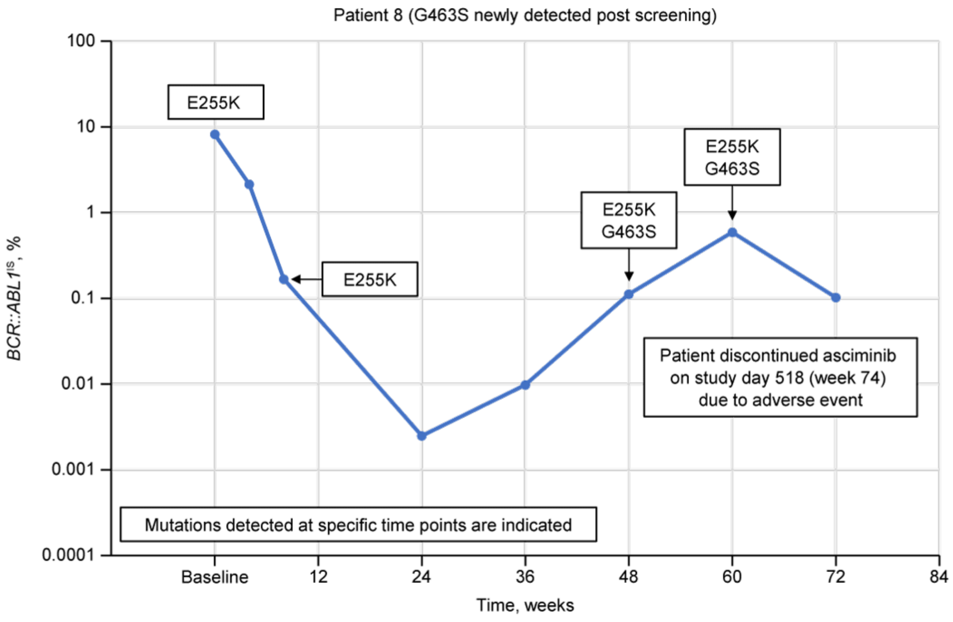

C Patient 9 (V289I newly detected post screening)

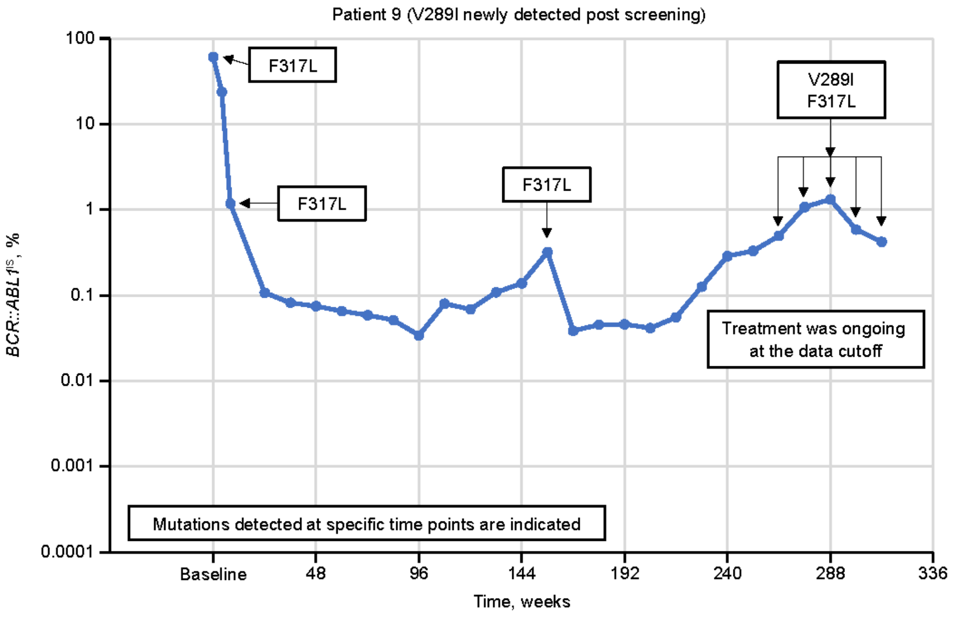

This patient's mutation status at weeks 228, 240, and 252 is unknown.

D Patient 10 (M244V newly detected post screening)

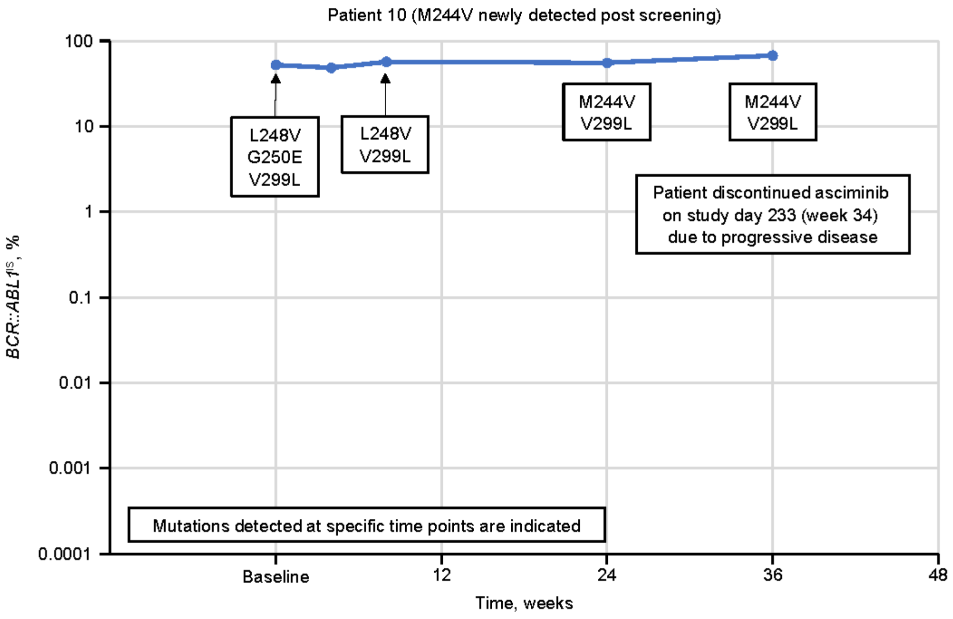

E Patient 11 (G463D newly detected post screening)

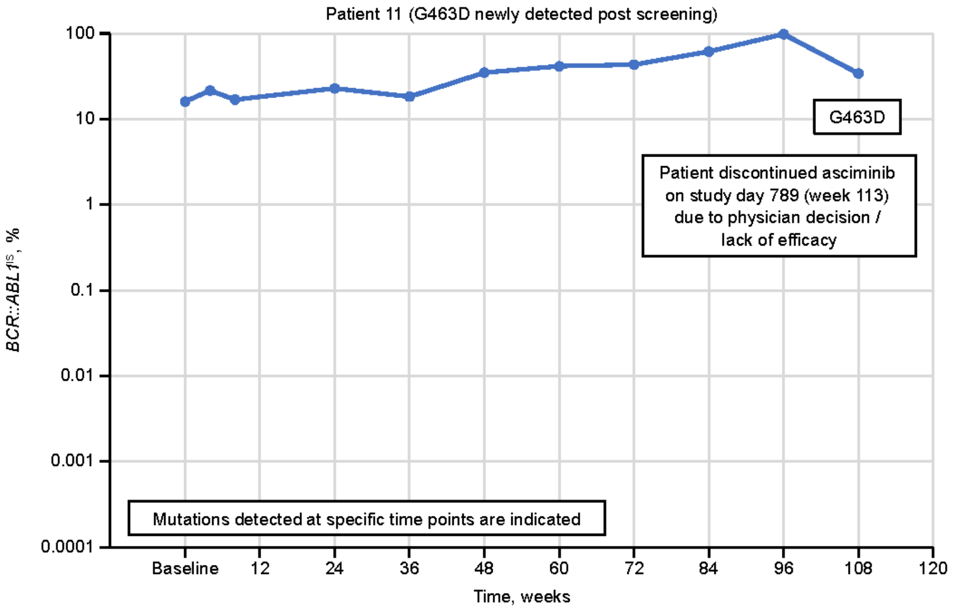

IS, International Scale.

[a] Mutation analysis was not comprehensively performed for all patients. Mutation analysis was performed using Sanger sequencing.

Supplemental Figure S7. EFS[a]

A. EFS for patients overall

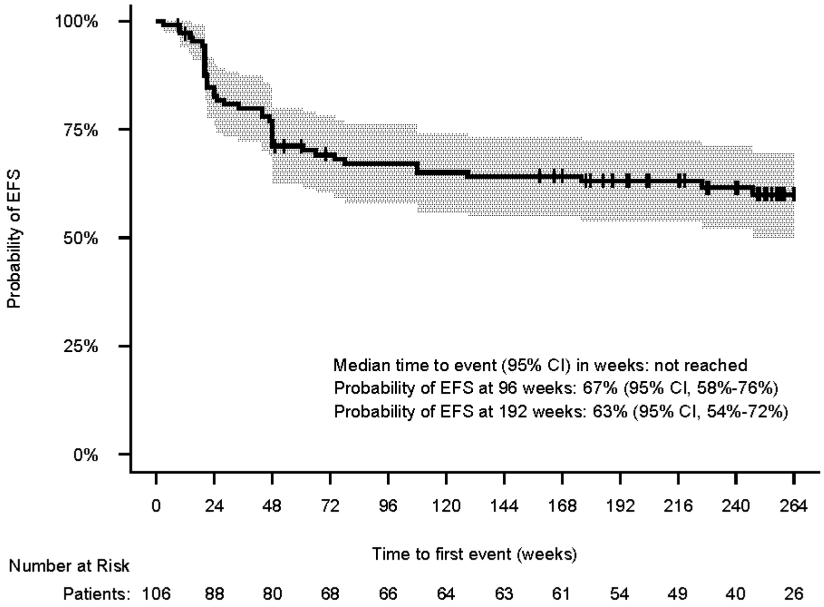

B. EFS by *BCR::ABL1*^{IS} at screening

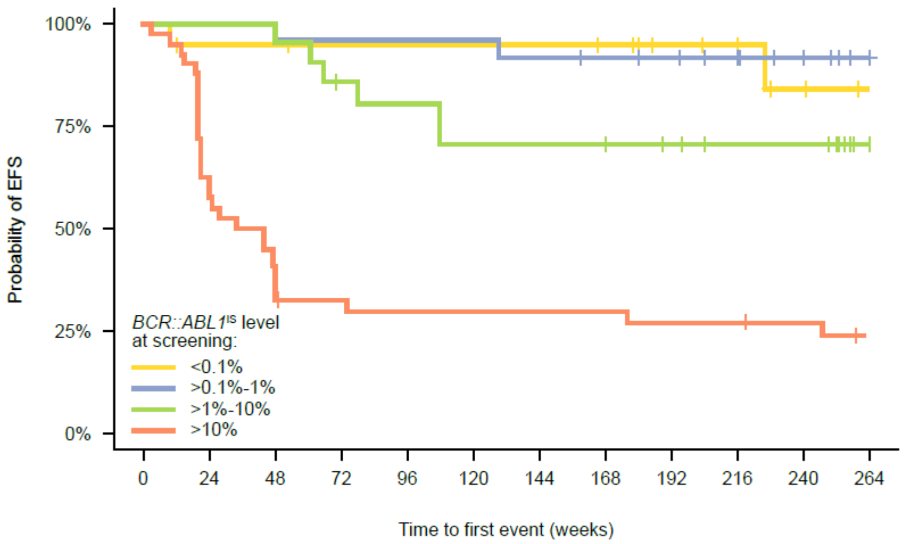

Number at Risk												
BCR::ABL1^{IS} <0.1%:	20	18	18	16	16	16	16	15	12	11	7	5
BCR::ABL1^{IS} >0.1%-1%:	24	24	24	23	23	23	22	21	20	18	14	10
BCR::ABL1^{IS} >1%-10%:	21	21	21	17	16	14	14	14	12	10	10	4
BCR::ABL1^{IS} >10%:	41	25	17	12	11	11	11	11	10	10	9	7

AE, adverse event; AP, accelerated phase; BC, blast crisis; EFS, event-free survival; IS, International Scale.

[a] EFS was estimated using the Kaplan-Meier method, with treatment discontinuation due to AEs, on-treatment progression to AP/BC, on-treatment death for any reason, *BCR::ABL1*^{IS} >10% at 6 months, and *BCR::ABL1*^{IS}>1% at ≥12 months considered events. Survival data were not collected after patients discontinued the study.
