# Supplementary material for: Asciminib monotherapy in patients with CML-CP without BCR::ABL1 T315I mutations treated with at least two prior TKIs: 4-year phase 1 safety and efficacy results
Source: Leukemia. 2023 Mar 22;37(5):1048–59. doi: 10.1038/s41375-023-01860-w (PMC10169635; doi:10.1038/s41375-023-01860-w)
Supplement: Supplementary file 2 — Supplementary Table S3 [file 41375_2023_1860_MOESM2_ESM.docx]

**Supplemental Table S3. *BCR::ABL1* mutations and MMR achievement^a^**

| Mutation detected at screening | MMR achieved at any time | Discontinued treatment (reason) | Mutation detected post screening |
| --- | --- | --- | --- |
| Patients with mutations detected at screening | | | |
| E255K | Yes | No | E225K |
| E255K | Yes | Yes (adverse event) | E255K and G463S^b,c^ |
| F317L | Yes | No | None |
| F317L | No | Yes (physician decision/  lack of efficacy) | F317L |
| F317L | Yes | No | F317L and V289I^b^ |
| G250E | Yes | No | G250E |
| G250E, L248V, and V299L | No | Yes (progressive disease) | L248V, M244V,^b^ and V299L |
| G250E and M244V | No | Yes (progressive disease) | M244V |
| L248V | No | Yes (adverse event) | L248V |
| M244V | No | Yes (progressive disease) | M244V |
| V299L | No | Yes (progressive disease) | I502L^b,c^ and V468F^b,c^ |
| Y253H | Yes | No | Y253H |
| Patients with mutations detected only post screening | | | |
| None | No | Yes (physician decision/  lack of efficacy) | G463D^b,c^ |

MMR, major molecular response (*BCR::ABL1*^IS^ ≤0.1% on the International Scale).

^a^ Postscreening mutation analysis was performed at the investigator’s discretion.

^b^ Mutation newly detected post screening.

^c^ *BCR::ABL1* myristoyl pocket mutation.
