# Supplementary material for: Asciminib monotherapy in patients with CML-CP without BCR::ABL1 T315I mutations treated with at least two prior TKIs: 4-year phase 1 safety and efficacy results
Source: Leukemia. 2023 Mar 22;37(5):1048–59. doi: 10.1038/s41375-023-01860-w (PMC10169635; doi:10.1038/s41375-023-01860-w)
Supplement: Supplementary file 3 — Supplementary Table S5 [file 41375_2023_1860_MOESM3_ESM.docx]

**Supplemental Table S5. Clinically important safety information: pancreatitis (clinical events)**

| **Patient no.** | **Age (years) /sex** | **Prior TKI** | **Preferred term** | **Asciminib starting dose, dose at event onset^a^/action taken with asciminib due to event** | **Study day on which event occurred** | **Baseline risk factors^b^ and relevant medical history** | **Patient’s status at cutoff date** |
| --- | --- | --- | --- | --- | --- | --- | --- |
| 1 | 60/Male | Nilotinib, imatinib, and dasatinib | Pancreatitis (grade 2; only radiological findings) | 40 mg twice daily, 80 mg twice daily as of study day 117/none | 148 | Acute pancreatitis | Discontinued as of study day 148 due to disease progression after receiving asciminib for 142 days |
| 2 | 59/Male | Nilotinib and dasatinib | Pancreatitis; 3 episodes (all grade 2) | 80 mg once daily, 80 mg once daily/treatment interrupted; 80 mg once daily/adjusted to 40 mg once daily; 40 mg once daily/none | 57, 127, and 297 | Obesity, HTN, diabetes type 2, hypertriglyceridemia, blood cholesterol increased, and increased lipase | Ongoing with asciminib 40 mg once daily |
| 3 | 54/Female^c^ | Imatinib and dasatinib | Pancreatitis; 4 episodes (all grade 3 except second episode [grade 2]) | 200 mg once daily, 200 mg once daily/treatment interrupted; 200 mg once daily/treatment adjusted to 120 mg once daily; 120 mg once daily/treatment adjusted to 40 mg once daily; 40 mg twice daily/treatment adjusted to 40 mg once daily | 6, 40, 82, and 811 | Cholelithiasis, cholecystectomy, hypercholesterolemia, hypertriglyceridemia, HTN, hyperglycemia (developed diabetes on day 56), alcohol consumption (may have contributed per investigator) | Ongoing with asciminib 40 mg once daily |
| 4 | 50/Male^d^ | Imatinib and dasatinib | Pancreatitis (grade 3) | 200 mg once daily, 200 mg once daily/treatment interrupted and then restarted at 120 mg once daily | 11 | Hypertriglyceridemia | Ongoing with asciminib 40 mg once daily |
| 5 | 58/Female | Imatinib and ponatinib | Pancreatitis (grade 3); chronic pancreatitis (grade 2) | 160 mg twice daily, 160 mg twice daily/treatment interrupted and then restarted at 120 mg twice daily; 120 mg twice daily/dose adjusted to 80 mg twice daily | 59 and 762 | Obesity, HTN, diabetes, and mesenteric artery thrombosis | Ongoing with asciminib 80 mg twice daily |
| 6 | 58/Male | Dasatinib, imatinib, and nilotinib | Pancreatitis (grade 2) | 150 mg twice daily, 150 mg twice daily/interrupted as of study day 156 and then discontinued | 155 | Social alcohol use, hyperlipidemia, hypertriglyceridemia, and hyperglycemia | Discontinued as of study day 195 due to pancreatic events, including enzyme elevations |
| 7 | 70/Male | Nilotinib, dasatinib, and bosutinib | Pancreatitis (grade 2) | 40 mg twice daily, 80 mg twice daily (as of study day 71)/treatment interrupted and then discontinued | 127 | HTN, pancreatic steatosis, and past episodes of amylase and lipase increase | Discontinued as of study day 149 due to pancreatic events |
| 8 | 53/Male | Nilotinib and dasatinib | Pancreatitis (grade 3) | 40 mg twice daily, 80 mg twice daily (as of study day 448)/treatment interrupted and then discontinued | 505 | Hypertrophic cardiomyopathy and pleural effusion | Discontinued as of study day 518 due to pancreatic events after receiving asciminib for 505 days |

HTN, hypertension; TKI, tyrosine kinase inhibitor.

^a^ Only the asciminib starting dose and dose at onset are reported; any additional dose levels are not reported here.

^b^ Baseline risk factors were not stringently collected as per protocol and were retrieved from general medical history as reported by investigators.

^c^ This patient also experienced grade 3 myocardial infarction and grade 2 angina pectoris (see **Supplemental Table S6**; second patient).

^d^ This patient also experienced grade 2 angina pectoris, grade 3 myocardial infarction, and grade 2 myocardial ischemia (see **Supplemental Table S6**; third patient).
