# Supplementary material for: Asciminib monotherapy in patients with CML-CP without BCR::ABL1 T315I mutations treated with at least two prior TKIs: 4-year phase 1 safety and efficacy results
Source: Leukemia. 2023 Mar 22;37(5):1048–59. doi: 10.1038/s41375-023-01860-w (PMC10169635; doi:10.1038/s41375-023-01860-w)
Supplement: Supplementary file 4 — Supplementary Table S6 [file 41375_2023_1860_MOESM4_ESM.docx]

**Supplemental Table S6. Clinically important safety information: AOEs^a^**

| **Age (years)/sex** | **Prior TKI** | **Preferred term** | **Asciminib starting dose, dose at event onset^b^/action taken with asciminib due to event** | **Study day on which event occurred** | **Baseline CV risk factors^c^ and relevant medical history** | **Patient’s status at cutoff date** |
| --- | --- | --- | --- | --- | --- | --- |
| 60/Male | Imatinib, nilotinib, dasatinib, and bosutinib | Cerebrovascular accident (grade 2) | 120 mg once daily/dose reduced to 80 mg once daily | 738 | Smoking, hyperlipidemia, HTN, obesity, and myocardial ischemia | Ongoing with asciminib at 80 mg once daily |
| 54/Female^d^ | Imatinib and dasatinib | Myocardial infarction (grade 3); angina pectoris (grade 2) | 200 mg once daily, 120 mg once daily as of study day 64 and 40 mg twice daily as of study day 101/treatment interrupted | 84 and 459 | Hyperlipidemia, hyperglycemia (diabetes diagnosed on day 72), HTN, and obesity | Ongoing with asciminib at 40 mg once daily |
| 50/Male^e^ | Imatinib and dasatinib | Angina pectoris (grade 2); myocardial infarction (grade 3); myocardial ischemia (grade 2) | 200 mg once daily, 80 mg once daily as of study day 29 and 40 mg once daily as of study day 256/treatment interrupted | 259 and 271 (for both myocardial infarction and ischemia events) | Hypertriglyceridemia and obesity | Ongoing with asciminib 40 mg once daily |
| 66/Female | Imatinib and nilotinib | Carotid artery stenosis (grade 2) | 120 mg once daily/dose reduced to 40 mg twice daily | 1 088 | HTN, hyperlipidemia, and rheumatoid arthritis | Ongoing with asciminib 40 mg twice daily |
| 65/Female^f^ | Imatinib, dasatinib, nilotinib, and bosutinib | Myocardial ischemia (grade 3) | 80 mg once daily and 200 mg once daily as of study day 418/none | 681 and 888 (2 episodes) | Hypercholesteremia and carotid artery stenosis | Died on study day 1 325 due to cardiac arrest contributed to by systemic scleroderma and ischemic heart disease |
| 49/Female | Imatinib, nilotinib, and dasatinib | Angina pectoris (grade 1) | 200 mg twice daily/none | 90 | Sjogren syndrome | Ongoing with asciminib at 80 mg twice daily |
| 46/Female | Imatinib, nilotinib, dasatinib, and bosutinib | Angina pectoris (grade 1) | 80 mg once daily and 40 mg once daily as of study day 15/none | 131 | Hyperlipidemia and obesity | Discontinued as of study day 760 due to thrombocytosis |
| 43/Male | Imatinib and nilotinib | Cerebellar infarction (grade 2) | 200 mg once daily and 40 mg once daily as of study day 435/treatment interruption | 496 | HTN, hypertriglyceridemia; of note, cerebellar infarction was associated with vertebral artery dissection, and the event was considered accidental | Discontinued as of study day 904 due to amylase and lipase increased |
| 70/Male | Imatinib, bosutinib, and dasatinib | Peripheral arterial occlusive disease (grade 3); arterial bypass occlusion (grade 3) | 20 mg twice daily/dose reduced to 20 mg once daily | 372 and 436 | HTN, diabetes mellitus II, coronary artery disease with 2 stents, hypertriglyceridemia, Sjogren syndrome | Died on study day 474 (>30 days after last dose) due to pneumonia aspiration |
| 86/Male^g^ | Imatinib, bosutinib, dasatinib, and ponatinib | Coronary artery disease (grade 3) | 80 mg once daily and 20 mg once daily as of study day 135/none | 659 | HTN, chronic heart failure, angina, and cerebral infarction | Discontinued as of study day 673 due to leukocytosis |

AOE, arterial occlusive event; CV, cardiovascular; HTN, hypertension; MedDRA, Medical Dictionary for Regulatory Activities; PT, preferred term; TKI, tyrosine kinase inhibitor.

^a^ AOEs were defined as grouped terms of 3 standardized MedDRA queries (ischemic central nervous system vascular conditions; ischemic heart disease [narrow]; embolic and thrombotic events, arterial). This search definition allowed for the exclusion of nonspecific events, which do not have a defined cardiovascular etiology, and allowed for the inclusion of PTs that are relevant to the cardiac and vascular system.

^b^ Only the asciminib starting dose and dose at onset are reported; any additional dose levels are not reported here.

^c^ Baseline CV risk factors were not stringently collected as per protocol and were retrieved from general medical history as reported by investigators.

^d^ This patient also experienced grade 2 and 3 pancreatitis (see **Supplemental Table S5**; third patient).

^e^ This patient also experienced grade 3 pancreatitis (see **Supplemental Table S5**; fourth patient).

^f^ This patient also experienced grade 4 cardiac failure acute and grade 3 cardiac failure acute-recurrent (see **Supplemental Table S7**; second patient).

^g^ This patient also experienced grade 3 cardiac failure (see **Supplemental Table S7**; last patient).
