# Supplementary material for: Asciminib monotherapy in patients with CML-CP without BCR::ABL1 T315I mutations treated with at least two prior TKIs: 4-year phase 1 safety and efficacy results
Source: Leukemia. 2023 Mar 22;37(5):1048–59. doi: 10.1038/s41375-023-01860-w (PMC10169635; doi:10.1038/s41375-023-01860-w)
Supplement: Supplementary file 5 — Supplementary Table S7 [file 41375_2023_1860_MOESM5_ESM.docx]

**Supplemental Table S7. Clinically important safety information: cardiac failure (clinical events)**

| **Age (years)/sex** | **Prior TKI** | **Preferred term** | **Asciminib starting dose, dose at event onset^a^/action taken with asciminib due to event** | **Study day on which event occurred** | **Baseline CV risk factors^b^ and relevant medical history** | **Patient’s status at cutoff date** |
| --- | --- | --- | --- | --- | --- | --- |
| 70/Male | Imatinib, nilotinib, dasatinib, and bosutinib | 1. Acute pulmonary edema (grade 3)  2. Pulmonary edema (grade 3) and cardiac failure congestive  (grade 3)  3. Cardiac failure congestive (grade 3) | 40 mg twice daily/dose interrupted | 1. 126  2. 164  3. 942 | Diabetes type 2, HTN, CKD, and dyslipidemia; atrioventricular block complete with cardiac pacemaker insertion | Discontinued as of study day 1 513 due to chronic kidney disease |
| 65/Female^c^ | Imatinib, dasatinib, nilotinib, and bosutinib | Cardiac failure acute (grade 4); cardiac failure acute (grade 3)-recurrent | 80 mg once daily and 200 mg once daily as of study day 418/none | 860 and 1 037 | Hyperlipidemia, carotid artery stenosis, and systemic scleroderma | Died on study day 1 325 due to cardiac arrest contributed to by systemic scleroderma and ischemic heart disease |
| 79/Female | Imatinib, nilotinib, dasatinib, and bosutinib | Pulmonary edema  (grade 1) | 120 mg once daily and 160 mg twice daily as of study day 919/none | 1 029 | Dyslipidemia, obesity, pulmonary HTN, HTN, and smoking | Ongoing with asciminib 20 mg twice daily (dose reduced due to lipase increased) |
| 61/Male | Nilotinib, dasatinib, and bosutinib | Cardiac failure congestive (grade 3) | 150 mg twice daily/temporarily interrupted | 1 291 | HTN, hyperlipidemia, obesity, and COPD with pulmonary HTN | Ongoing with asciminib 160 mg twice daily |
| 59/Female | Imatinib, nilotinib, dasatinib, and bosutinib | Cardiac failure congestive (grade 3) | 80 mg once daily/temporarily interrupted | 981 | Severe obesity, hypertrophic cardiomyopathy, HTN, and hyperlipidemia | Ongoing with asciminib 80 mg once daily |
| 30/Male | Nilotinib and dasatinib | Ejection fraction decreased (grade 2) | 40 mg twice daily/none | 15 | None | Ongoing with asciminib 40 mg twice daily |
| 86/Male^d^ | Imatinib, bosutinib, dasatinib, and ponatinib | Cardiac failure (grade 3) | 80 mg once daily and 20 mg once daily as of study day 135/none | 651 | Chronic cardiac failure, HTN, and angina pectoris | Discontinued as of study day 673 due to leukocytosis |

CKD, chronic kidney disease; COPD, chronic obstructive pulmonary disease; CV, cardiovascular; HTN, hypertension; TKI, tyrosine kinase inhibitor.

^a^ Only the asciminib starting dose and dose at onset are reported; any additional dose levels are not reported here.

^b^ Baseline CV risk factors were not stringently collected as per protocol and were retrieved from general medical history as reported by investigators.

^c^ This patient also experienced grade 3 myocardial ischemia (see **Supplemental Table S6**; fifth patient).

^d^ This patient also experienced grade 3 coronary artery disease (see **Supplemental Table S6**; last patient).
