# Supplementary material for: Asciminib monotherapy in patients with CML-CP without BCR::ABL1 T315I mutations treated with at least two prior TKIs: 4-year phase 1 safety and efficacy results
Source: Leukemia. 2023 Mar 22;37(5):1048–59. doi: 10.1038/s41375-023-01860-w (PMC10169635; doi:10.1038/s41375-023-01860-w)
Supplement: Supplementary file 6 — Supplementary Table S8 [file 41375_2023_1860_MOESM6_ESM.docx]

**Supplemental Table S8. Criteria for defining DLTs^a^**

| **Toxicity** | **Any of the following criteria for which relationships to study treatment could not be ruled out** |
| --- | --- |
| Hematology (CML CP or AP) | - CTCAE grade 4 neutropenia (ANC <0.5 × 10^9^/L) lasting >5 days - CTCAE grade 4 thrombocytopenia (platelets <25 × 10^9^/L) or grade 3 thrombocytopenia with bleeding - CTCAE grade 4 febrile neutropenia (fever >38.3°C) - CTCAE grade 4 anemia unexplained by underlying disease - CTCAE grade 3 anemia was not considered a DLT unless judged to be a hemolytic process secondary to study treatment |
| Hematology (Ph+ ALL/CML BP) | Since marrow aplasia is an expected consequence of Ph+ ALL therapy, only persistent pancytopenia that continued for ≥42 days and was not related to leukemic infiltration was considered a DLT. Bone marrow evaluation may have been required to determine whether marrow aplasia was due to leukemia |
| Gastrointestinal | - CTCAE grade ≥3 vomiting or nausea uncontrolled by medical management - CTCAE grade ≥3 diarrhea despite optimal antidiarrheal treatment |
| Pancreatic | - Asymptomatic CTCAE grade 3 or 4 elevation of amylase (of pancreatic origin) or lipase, or asymptomatic radiologic pancreatitis (grade 2 pancreatitis) - Grade ≥3 pancreatitis |
| Pleural effusion | CTCAE grade ≥2 requiring intervention |
| Other AEs | - CTCAE grade ≥3 AEs of any other type, except for the exclusions noted below - Any other clinically significant toxicity that the investigator and sponsor considered dose limiting |
| Exceptions to the DLT criteria | - ≤3 days of CTCAE grade 3 fatigue - CTCAE grade 3 or 4 lymphopenia or alopecia of any grade - Grade 3 electrolyte abnormalities that could be corrected in ≤7 days and which the investigator considered not clinically important |

AE, adverse event; ALL, acute lymphoblastic leukemia; ANC, absolute neutrophil count; AP, accelerated phase; BP, blast phase; CML, chronic myeloid leukemia; CP, chronic phase; CTCAE, Common Terminology Criteria for Adverse Events; DLT, dose-limiting toxicity; Ph+, Philadelphia chromosome positive.

^a^ CTCAE version 4.03 was used for grading all AEs and laboratory abnormalities.
