# Supplementary material for: Asciminib monotherapy in patients with CML-CP without BCR::ABL1 T315I mutations treated with at least two prior TKIs: 4-year phase 1 safety and efficacy results
Source: Leukemia. 2023 Mar 22;37(5):1048–59. doi: 10.1038/s41375-023-01860-w (PMC10169635; doi:10.1038/s41375-023-01860-w)
Supplement: Supplementary file 7 — Supplementary Table S9 [file 41375_2023_1860_MOESM7_ESM.docx]

**Supplemental Table S9. Summary of PK parameters for asciminib by day and treatment^a^**

| **Treatment** | **Statistics** | **AUC_last_**  **(ng.hr/mL)** | **AUC_tau_**  **(ng.hr/mL)** | **C_max_**  **(ng/mL)** | **T_max_**  **(h)** | **C_trough_ (ng/mL)** | **Racc** |
| --- | --- | --- | --- | --- | --- | --- | --- |
| **Cycle 1, day 1** |  |  |  |  |  |  |  |
| Asciminib 40 mg twice daily (n=32) | n | 30 |  | 30 | 30 |  |  |
|  | Mean (SD) | 2 671.14 (1 729.55) |  | 653.30 (468.04) |  |  |  |
|  | CV% mean | 64.75 |  | 71.64 |  |  |  |
|  | Geo-mean | 2 246.76 |  | 536.85 |  |  |  |
|  | CV% geo-mean | 69.25 |  | 74.29 |  |  |  |
|  | Median | 2 182.51 |  | 555.50 | 2.10 |  |  |
|  | Min; max | 287.38; 9 417.52 |  | 61.90; 2 610.00 | 1.95; 5.62 |  |  |
| Asciminib 80 mg once daily (n=18) | n | 18 |  | 18 | 18 |  |  |
|  | Mean (SD) | 11 556.4 (4160.31) |  | 1 252.78 (448.26) |  |  |  |
|  | CV% mean | 36.00 |  | 35.78 |  |  |  |
|  | Geo-mean | 10 752.4 |  | 1 157.54 |  |  |  |
|  | CV% geo-mean | 42.90 |  | 46.46 |  |  |  |
|  | Median | 1 1434.4 |  | 1 275.00 | 2.06 |  |  |
|  | Min; max | 4 232.60; 18 693.9 |  | 445.00; 1 850.00 | 1.13; 6.00 |  |  |
| Asciminib 200 mg twice daily (n=62) | n | 61 |  | 61 | 61 |  |  |
|  | Mean (SD) | 15 658.5 (5 120.07) |  | 3 646.07 (1 160.51) |  |  |  |
|  | CV% mean | 32.70 |  | 31.83 |  |  |  |
|  | Geo-mean | 14 869.9 |  | 3 464.70 |  |  |  |
|  | CV% geo-mean | 33.42 |  | 33.60 |  |  |  |
|  | Median | 14 657.9 |  | 3 460.00 | 2.03 |  |  |
|  | Min; max | 7 595.05; 28 751.9 |  | 1 390.00; 6 840.00 | 0.95; 7.28 |  |  |
| **Cycle 2, day 1** |  |  |  |  |  |  |  |
| Asciminib 40 mg twice daily (n=32) | n | 30 | 23 | 30 | 30 | 30 | 15 |
|  | Mean (SD) | 4 372.10 (1 832.70) | 5 776.50 (2 438.55) | 873.37 (369.08) |  | 308.01 (161.60) | 1.90 (1.28) |
|  | CV% mean | 41.92 | 42.21 | 42.26 |  | 52.46 | 67.49 |
|  | Geo-mean | 3 967.03 | 5 262.32 | 793.26 |  | 262.50 | 1.65 |
|  | CV% geo-mean | 49.64 | 48.49 | 48.92 |  | 67.53 | 56.82 |
|  | Median | 4 573.25 | 5 825.18 | 910.00 | 2.01 | 263.50 | 1.59 |
|  | Min; max | 1 439.44; 8 753.69 | 1 750.80; 12 259.6 | 300.00; 1 590.00 | 1.00; 6.00 | 59.20; 633.00 | 0.48; 6.21 |
| Asciminib 80 mg once daily (n=18) | n | 17 | 17 | 17 | 17 | 16 | 16 |
|  | Mean (SD) | 15 530.4 (4 076.54) | 15 632.5 (4 070.54) | 1 825.88 (422.42) |  | 207.50 (84.36) | 1.33 (0.27) |
|  | CV% mean | 26.25 | 26.04 | 23.14 |  | 40.66 | 20.40 |
|  | Geo-mean | 15 001.3 | 15 112.4 | 1 780.98 |  | 193.26 | 1.30 |
|  | CV% geo-mean | 28.27 | 27.85 | 23.34 |  | 39.58 | 20.29 |
|  | Median | 14 679.6 | 14 967.6 | 1 730.00 | 2.00 | 174.50 | 1.29 |
|  | Min; max | 8 260.88; 22 243.9 | 8 512.36; 22 395.7 | 1 190.00; 2 840.00 | 0.95; 4.10 | 124.00; 393.00 | 0.89; 1.99 |
| Asciminib 200 mg twice daily (n=62) | n | 54 | 34 | 54 | 54 | 55 | 28 |
|  | Mean (SD) | 32 409.0 (14 072.2) | 40 638.8 (18 473.5) | 6 069.44 (2 447.33) |  | 3 137.04 (1 899.00) | 1.96 (0.39) |
|  | CV% mean | 43.42 | 45.46 | 40.32 |  | 60.53 | 19.98 |
|  | Geo-mean | 29 924.6 | 37 547.0 | 5 641.84 |  | 2 715.38 | 1.92 |
|  | CV% geo-mean | 41.27 | 41.00 | 39.86 |  | 57.65 | 19.48 |
|  | Median | 28 141.3 | 37 092.1 | 5 335.00 | 2.00 | 2 500.00 | 1.89 |
|  | Min; max | 10 144.6; 84 207.8 | 13 473.3; 119 753 | 2 000.00; 15 200.0 | 0.90; 7.03 | 527.00; 11 200.0 | 1.34; 2.92 |

AP, accelerated phase; AUC_last_, area under the curve up to the last measurable concentration; AUC_tau_, area under the curve during a dosing interval; CML, chronic myeloid leukemia; C_max_, maximum (peak) concentration of drug; CP, chronic phase; C_trough_, minimum concentration of drug; CV%, coefficient of variation; Geo-mean, geometric mean; PK, pharmacokinetics; Racc, accumulation ratio; SD, standard deviation; T_max_, time to reach maximum plasma concentration.

^a^ Data are based on the PK analysis set of the population of patients with CML in CP or AP, regardless of T315I status, who received asciminib monotherapy. CV% = SD/mean*100. CV% geo-mean = sqrt (exp (variance for log transformed data)−1)*100. Accumulation half-life (T1/2, acc) = ln(2)*tau/ln(Racc/(Racc−1)).
